# A Novel Amino Acid‐Related Gene Signature Predicts Overall Survival in Patients With Hepatocellular Carcinoma

**DOI:** 10.1002/cnr2.2131

**Published:** 2024-07-23

**Authors:** Shuyi Wang, Hong Huang, Xingwang Hu, Meifang Xiao, Kaili Yang, Haiyan Bu, Yupeng Jiang, Zebing Huang

**Affiliations:** ^1^ Hunan Key Laboratory of Viral Hepatitis, Department of Infectious Diseases, Nation Clinical Research Center for Geriatric Disorders Xiangya Hospital, Central South University Changsha Hunan China; ^2^ Guilin Medical University Guilin China; ^3^ Department of Health Management Center Xiangya Hospital, Central South University Changsha China; ^4^ Department of Oncology The Second Xiangya Hospital, Central South University Changsha China

**Keywords:** biomarkers, hepatocellular carcinoma, metabolic studies, prognosis

## Abstract

**Background and Aims:**

Hepatocellular carcinoma (HCC) is an extremely harmful malignant tumor in the world. Since the energy metabolism and biosynthesis of HCC cells are closely related to amino acids, it is necessary to further explore the relationship between amino acid‐related genes and the prognosis of HCC to achieve individualized treatment. We herein aimed to develop a prognostic model for HCC based on amino acid genes.

**Methods:**

In this study, RNA‐sequencing data of HCC patients were downloaded from the TCGA‐LIHC cohort as the training cohort and the GSE14520 cohort as the validation cohort. Amino acid‐related genes were derived from the Molecular Signatures Database. Univariate Cox and Lasso regression analysis were used to construct an amino acid‐related signature (AARS). The predictive value of this risk score was evaluated by Kaplan–Meier (K–M) curve, receiver operating characteristic (ROC) curve, univariate and multivariate Cox regression analysis. Gene set variation analysis (GSVA) and immune characteristics evaluation were used to explore the underlying mechanisms. Finally, a nomogram was established to help the personalized prognosis assessment of patients with HCC.

**Results:**

The AARS comprises 14 amino acid‐related genes to predict overall survival (OS) in HCC patients. HCC patients were divided into AARS‐high group and AARS‐low group according to the AARS scores. The K–M curve, ROC curve, and univariate and multivariate Cox regression analysis verified the good prediction efficiency of the risk score. Using GSVA, we found that AARS variants were concentrated in four pathways, including cholesterol metabolism, delayed estrogen response, fatty acid metabolism, and myogenesis metabolism.

**Conclusion:**

Our results suggest that the AARS as a prognostic model based on amino acid‐related genes is of great value in the prediction of survival of HCC, and can help improve the individualized treatment of patients with HCC.

## 1 Introduction

Hepatocellular carcinoma (HCC) is a highly prevalent and extremely harmful malignancy in the world, accounting for ~90% of liver cancer cases [1]. It is estimated that by 2040, 1.4 million new cases of liver cancer will be diagnosed with an increase of 55% over 2020, and 1.3 million people will die of liver cancer with the mortality rate of liver cancer increasing by 56.4% according to the mortality rate of 2020 [2]. Because of the high incidence and mortality, timely prognostic assessment and intervention are essential for patients diagnosed with HCC.

At present, Barcelona Clinic Liver Cancer (BCLC) algorithm is recognized as the most widely used staging system, underlying the potential prognosis of HCC by measuring tumor load, tumor‐related symptoms, and evaluation of the underlying liver [3]. Current treatments including hepatectomy, tumor ablation, liver transplantation, transarterial therapies, chemotherapy, radiotherapy, targeted and immune therapies, and the combination mode of topical treatment and systemic drugs have been recommended in recent years [4, 5]. However, the survival rate of liver cancer has barely improved over the past decade [6]. Moreover, even if the patient is in the early diagnosis and early intervention, there is still a possibility of poor prognosis occurring due to the heterogeneity of HCC leading to drug resistance and distant metastasis [7, 8]. Hence, traditional tumor classification, staging, and grading systems can not satisfy the need for individualized tumor treatment. To achieve accurate prediction, stratification, and personalized treatment of HCC patients, it is necessary to strengthen the research of prognosis classification.

Metabolic reprogramming is one of the hallmarks of HCC [9, 10]. The traditional view is that metabolic reprogramming in tumors is mainly focused on glucose, but with the gradual deepening of the exploration of tumor metabolism, metabolic reprogramming is no longer just a synonym for the Warburg effect [11, 12, 13]. Current studies have found that in addition to glucose metabolic dysregulation, amino acid metabolic dysregulation is an indispensable factor in tumor proliferation, invasion, and metastasis [14].

Amino acids are involved in energy generation and biosynthesis of tumor cells, and more studies support that amino acid function disorders are crucial in the development of HCC. A strong association has been found between the risk of HCC and circulating levels of several aromatic, branched, glycogenic amino acids and bioamines [15]. It has been discovered that the development and growth of HCC are promoted by the loss of catabolism of branched‐chain amino acids, and the inhibition degree of the catabolism enzyme is highly correlated with the aggressiveness of the tumor [16]. The overexpress of glutaminase 1 has been confirmed to promote HCC cell proliferation with involved the AKT/GSK3β/CyclinD1 pathway [17]. However, the metabolic reprogramming and related pathways of amino acid in HCC have not been systematically analyzed, and amino acid‐related signatures for the prognosis of HCC lack relevant studies.

To fill this gap in research, we composed an amino acid‐related signature and established an AARS formula for HCC patients that can predict prognosis independently with validation from various perspectives. Moreover, we combined AARS with the clinical parameters of HCC patients to construct a nomogram for clinical administration for HCC patients. These findings indicated that AARS might be a potential prognostic predictor of overall survival (OS) in HCC patients, and promoted a comprehensive understanding of the features of amino acid‐related genes in HCC.

## 2 Materials and Methods

### 2.1 Dataset Collection

The 424 HCC patients' RNA‐sequencing data and relevant clinical data of the TCGA‐LIHC dataset, including 50 adjacent nontumor samples and 374 tumor samples, were obtained from The Cancer Genome Atlas (TCGA) database (https://portal.gdc.cancer.gov/). After excluding normal samples and those without survival data, 371 tumor samples were selected. The GSE14520 dataset with samples lacking survival data removed was accessed from Gene Expression Omnibus (GEO) database (https://www.ncbi.nlm.nih.gov/geo/). Amino acid‐related genes were composed of amino acid and derivative metabolic process, amide biosynthetic process, amine metabolic process, amine transport, amino acid activation, amino acid transmembrane transport, amino acid transport, cellular amide metabolic process, and amide binding in Molecular Signatures Database (https://www.gsea‐msigdb.org/) [18].

### 2.2 Genetic Screening

A total of 1851 amino acid‐related genes were obtained to form a gene set (Table S1), and 1845 genes of the gene set were extracted in the TCGA‐LIHC cohort. Differential gene expression analysis in the TCGA‐LIHC cohort was performed using the “limma” R package and differentially expressed genes (DEGs) were selected with |logFC| >1.0 and *p* < 0.05 [19]. The DEGs in the TCGA‐LIHC cohort were intersected with the GSE14520 cohort to obtain coexpress genes by using the “sva” R package, and the combat function was applied to remove the batch effects.

### 2.3 Construction and Verification of AARS

First, univariate Cox regression analysis based on the coexpress genes was conducted to screen prognostic‐related DEGs in the TCGA‐LIHC training cohort. Then, Lasso Cox regression analysis was employed to narrow the number of genes to reduce the risk of overfitting and construct AARS. The AARS score for each HCC patient was calculated as follows:

$$\text{AARS}=\sum_{i=1}^{n}{\text{coef}}_{i}\times \exp r_{i}.$$

${\text{Coef}}_{i}$ represents the corresponding coefficient of each gene, and $\exp r_{i}$ represents the expression of each gene. Univariate and multivariate Cox regression analyses were used to evaluate whether AARS was an independent prognostic factor for HCC patients by comparing AARS with clinical characteristics such as age, gender, grade, and stage (*p* < 0.05). We divided the samples into the AARS‐high group and AARS‐low group according to the optimal cutoff value of the AARS score, which was calculated by the “survminer” R package. Survival curves between the AARS‐high and AARS‐low groups were plotted by “survminer” and “survival” R packages. The “survival ROC” R package was applied to perform time‐dependent ROC curve analysis to evaluate the performance of AARS. We verified the robustness of AARS with the GSE14520 cohort as the validation group.

### 2.4 Functional Enrichment Analysis

We conduct differential analysis between AARS‐high and AARS‐low groups in the TCGA‐LIHC cohort (*p* < 0.05, LogFC>| ± 1|), and detected a total of 1150 DGEs. The “GSVA” [20], “msigdbr” R [21], and “limma” packages were applied to discern differential enrichment pathways between AARS‐high and AARS‐low groups. The results were visualized by “ggplot2.”

### 2.5 Comprehensive Analysis of Immune Checkpoints, TMB, and PD‐L1

In the TCGA cohort, 22 immune cell infiltration results were obtained using “e1071,” “preprocessCore,” “limma” R package, and CIBERSORT, and visualized by “ggplot2” and “tidyr” R package [22, 23]. The correlation between the AARS and 22 immune cells was demonstrated and visualized by “corrplot” R package. To evaluate the potential benefit of immune checkpoint inhibitors (ICI) treatment, we compared immune checkpoint, tumor mutational burden (TMB), and programmed cell death‐ligand 1 (PD‐L1) between AARS‐high and AARS‐low groups by “ggpubr” R package.

### 2.6 Subgroup Analysis

The clinical features of HCC patients including age, gender, grade, and stage were downloaded from the TCGA database. The subgroup analysis based on different clinical features was performed by “survival” and “survminer” R package.

### 2.7 Nomogram Construction

To provide each HCC patient with an accurate digital survival risk, a predictive nomogram model was constructed to predict OS of HCC patients [24]. The calibration performance of nomograms was assessed by the calibration curve. The construction of nomogram model and calibration plots was performed using “rsm” R package.

### 2.8 Statistical Analysis

Statistical analyses were performed using R (version 4.1.0) software. Wilcoxon test was used to study the difference in immune infiltrating cell composition, and the log‐rank test was used to analyze Kaplan–Meier survival between groups. *p* < 0.05 was considered significant.

## 3 Results

### 3.1 Identification of Differential Amino Acid‐Related Gene Sets

**FIGURE 1:**
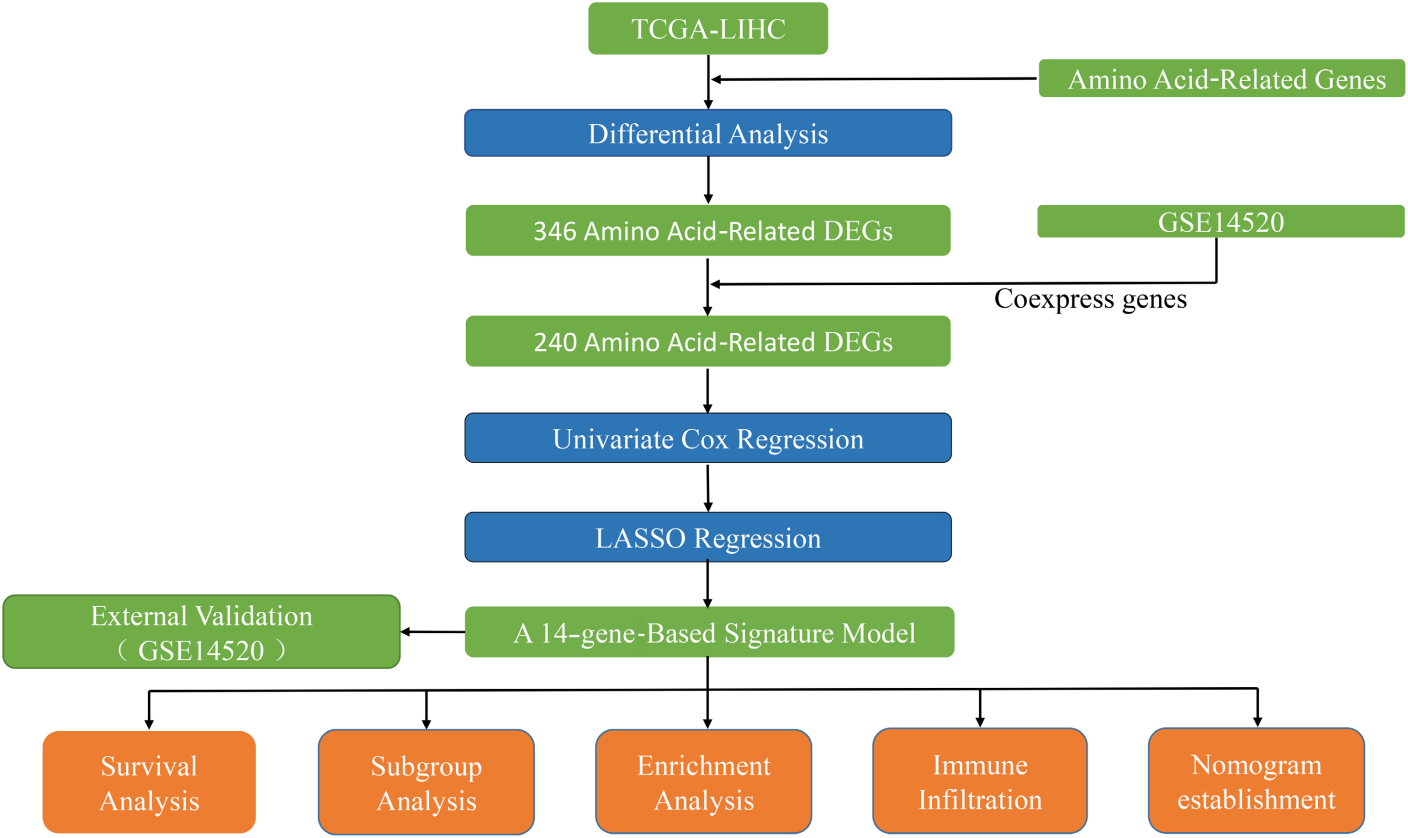
The overall workflow of this research.

**FIGURE 2:**
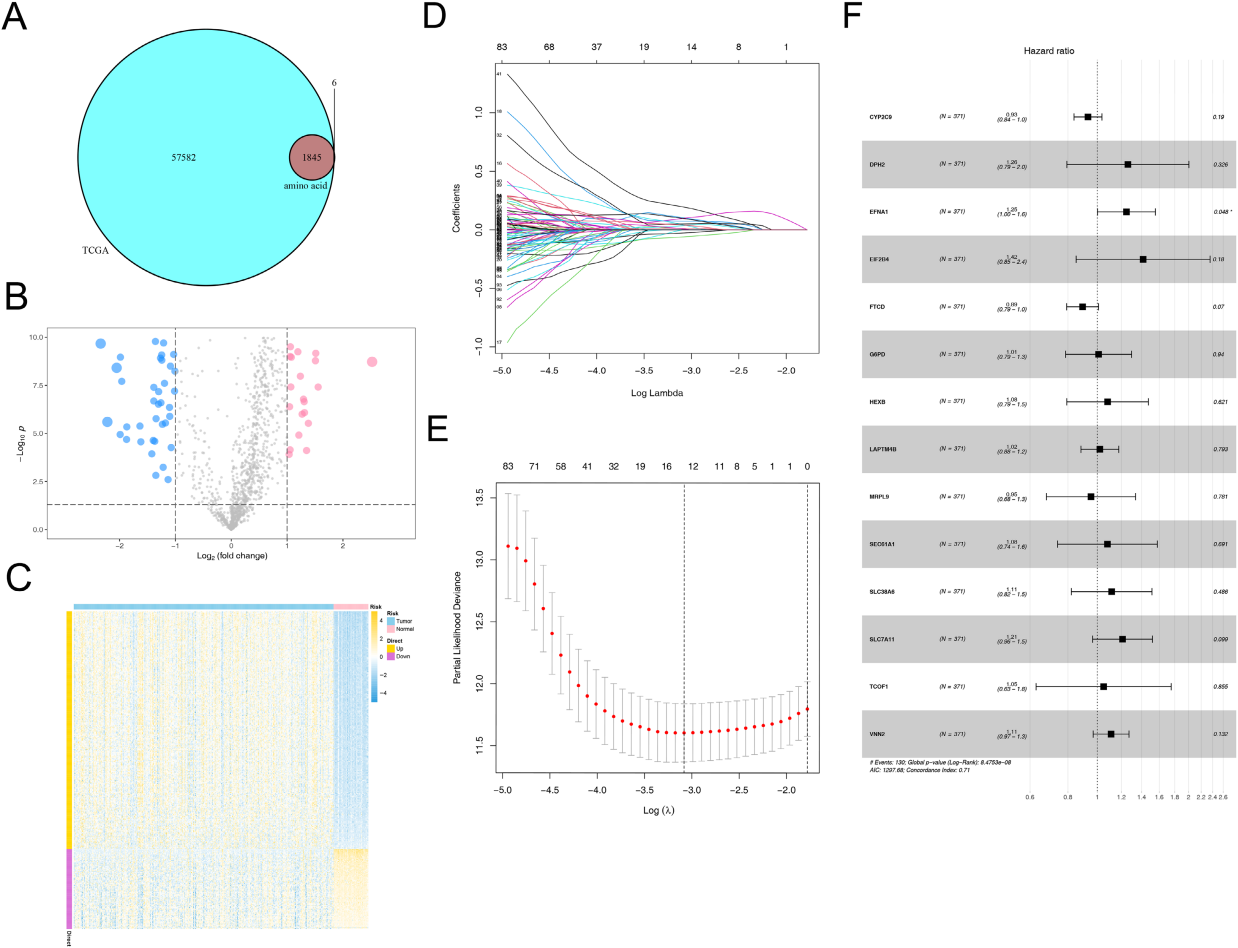
Identification of amino acid‐related DEGs in HCC. (A) The volcano plot of 240 amino acid‐related DEGs. (B) The heatmap of 240 amino acid‐related DEGs. (C, D) Lasso regression analysis. (E) Univariate Cox regression of 14 amino acid‐related DEGs associated with OS.

To present our study more intuitively and clearly, a flowchart was drawn to describe the process of prognostic model development and validation (Figure 1). Differential analysis of amino acid‐related genes in tumor and nontumor tissues in the TCGA‐LIHC cohort identified 340 DEGs associated with HCC prognosis. A total of 240 coexpressed genes were obtained by taking the intersection with the GSE14520 validation set (Figure 2A,B). The expression of most DEGs in tumor tissues is upregulated.

### 3.2 Establishment and Validation of the AARS Subgroups

**FIGURE 3:**
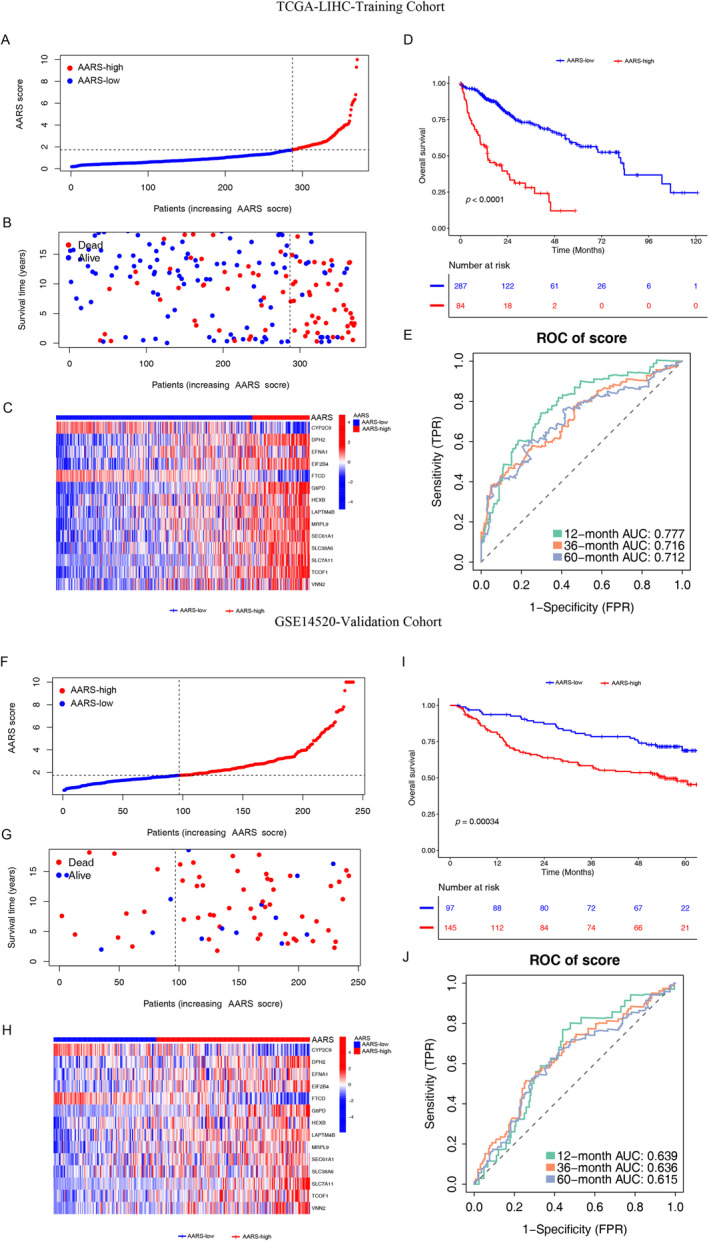
Characteristics and prognostic analysis of the AARS score based on 14 amino acid‐related genes. (A) Risk‐score distribution in the training cohort. (B) The survival status of patients in the AARS‐high and AARS‐low groups in the training cohort. (C) Heatmap of the expression of the amino acid‐related gene signatures in the AARS‐high and AARS‐low groups in the training cohort. (D) The K–M curves of the AARS‐high and AARS‐low groups in the training cohort. (E) The ROC curves of the prognostic performance of AARS score in the training cohort. (F) Risk‐score distribution in the validation cohort. (G) The survival status of patients in the AARS‐high and AARS‐low groups in the validation cohort. (H) Heatmap of the expression of the amino acid‐related gene signatures in the AARS‐high and AARS‐low groups in the validation cohort. (I) The K–M curves of the AARS‐high and AARS‐low groups in the validation cohort. (J) The ROC curves of the prognostic performance of the AARS score in the validation cohort.

Based on 240 DEGs, a total of 83 independent prognostic genes were selected as candidate genes by univariate Cox regression analysis. We further narrowed the number of variables and finally identified 14 genes to construct the risk score by Lasso–Cox regression analysis (Figure 2C,D). Among the 14 DEGs, 3 genes (CYP2C9, FTCD, and MRPL9) were protective factors with HR less than 1, while the other 11 genes (DPH2, EFNA1, EIF2B4, G6PD, HEXB, LAPTM4B, SEC61A1, SLC38A6, SLC7A11, TCOF1, and VNN2) were risk factors with HR more than 1 for HCC patients (Figure 2E). Then, a risk score for each patient was calculated based on the formula: AARS = G6PD ×  0.009569 + LAPTM4B × 0.019248 + TCOF1 × 0.047967 + SEC61A1 × 0.076787 + HEXB × 0.07803 + VNN2 × 0.104436 + SLC38A6 × 0.108746 + SLC7A11 × 0.191417 + EFNA1 × 0.221362 + DPH2 × 0.231957 + EIF2B4 × 0.347571 − FTCD × 0.11171 − CYP2C9 × 0.07101 − MRPL9 × 0.04807, and shown on a risk score distribution plot (Figure 3A,F). The distribution plot reflected the survival time and the AARS scores in HCC patients could be negatively correlated (Figure 3B,G). The expression of CYP2C9, FTCD, and MRPL9 decreased with the increase in AARS scores (Figure 3C,H). Consequently, we used the median AARS score as the optimal cutoff value to divide HCC patients into AARS‐high and AARS‐low groups, and K–M curves of survival was shown in Figure 3D,I. Patients with a high AARS score had significantly worse OS than patient with a low AARS score in TCGA‐LIHC cohort (*p* < 0.0001, log‐rank test). To assess the prognosis formula's prediction efficiency, the AUC values for predicting the 1‐, 3‐, and 5‐year OS were calculated as 0.777, 0.716, and 0.712, respectively, in TCGA‐LIHC cohort (Figure 3E). The same method obtained similar results for external validation on the GSE14520 cohort (Figure 3J).

### 3.3 Correlation Analysis Between the AARS Subgroups and Clinical Characteristics of HCC Patients

**FIGURE 4:**
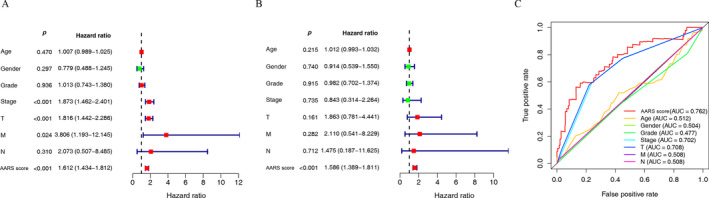
Comparison between AARS score and traditional models (including age, gender, grade, pathologic stage, T stage, N stage, and M stage). (A) Univariate Cox analysis of AARS score and clinical factors in TCGA‐LIHC cohort. (B) Multivariate Cox analysis of AARS score and clinical factors in TCGA‐LIHC cohort. (C) The ROC curves of AARS score and clinical factors for accuracy evaluation.

**FIGURE 5:**
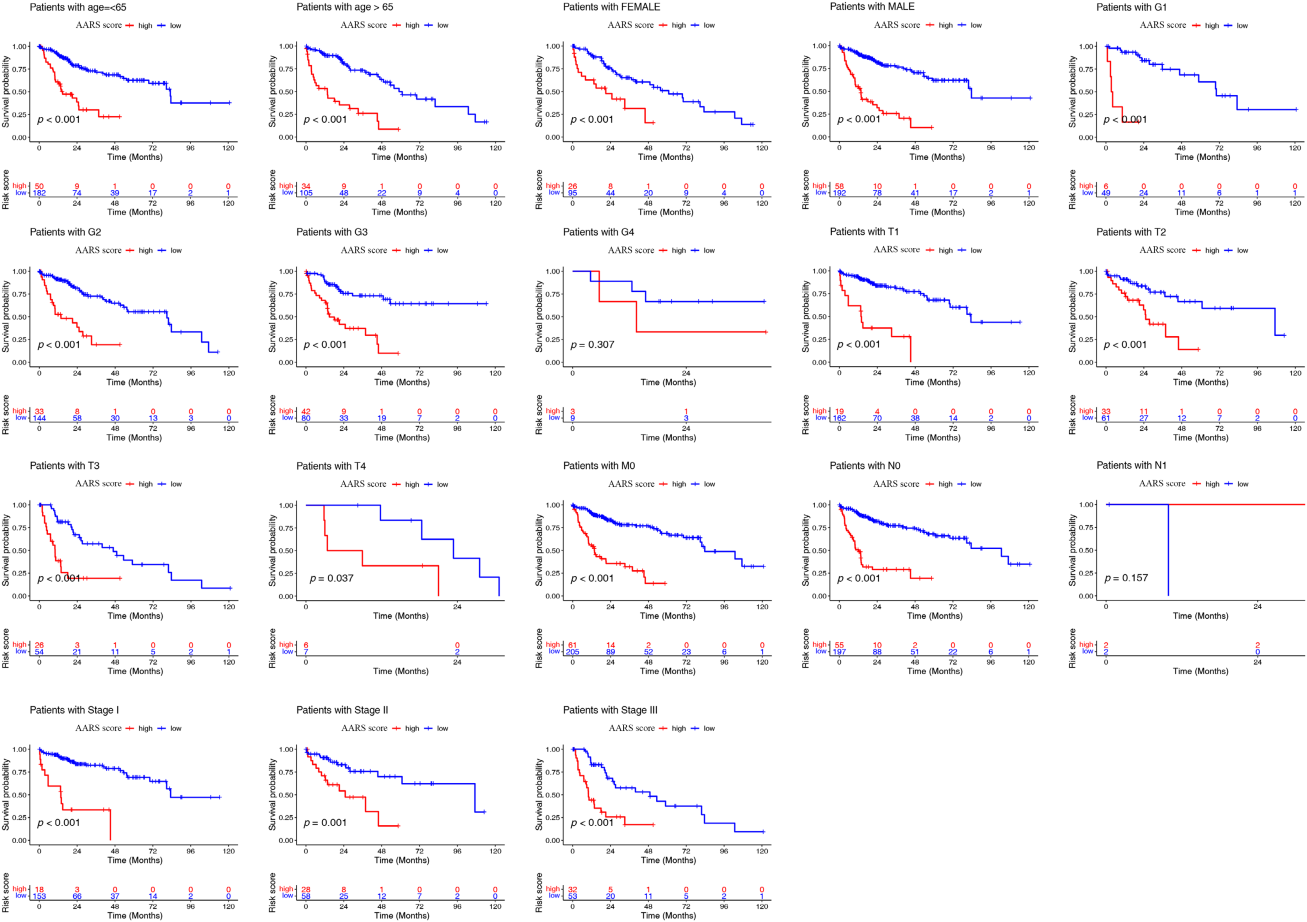
The overall survival differences between the AARS‐high and AARS‐low groups under the conditions of classifying patients by clinical features (including age, gender, grade, pathologic stage, T stage, N stage, and M stage).

To validate the independent prognostic value of AARS, we conducted univariate and multivariate Cox regression analyses of clinical factors and the AARS in the TCGA‐LIHC cohort. The pathological stage, T stage, M stage, and AARS were associated with OS of HCC patients through univariate Cox regression (Figure 4A). Both univariate and multivariate Cox regression demonstrated that the AARS was an independent prognostic factor for OS of HCC patients (Figure 4A,B). We conducted accuracy evaluation of different prognostic factors by ROC curve analysis. The AUC values of the AARS score, pathologic stage, and T stage were 0.762, 0.702, and 0.708, respectively (Figure 4C). Considering that HCC patients have different clinical characteristics that can affect their prognosis, we conducted a subgroup analysis of AARS‐high and AARS‐low groups according to different clinical features, including age, gender, grade, pathologic stage, T stage, M stage, and N stage (Figure 5). Patients in the AARS‐high group with different clinical features except G4 and N1 had poorer survival than those in the AARS‐low group.

### 3.4 Functional Enrichment Analysis in the AARS Subgroups

**FIGURE 6:**
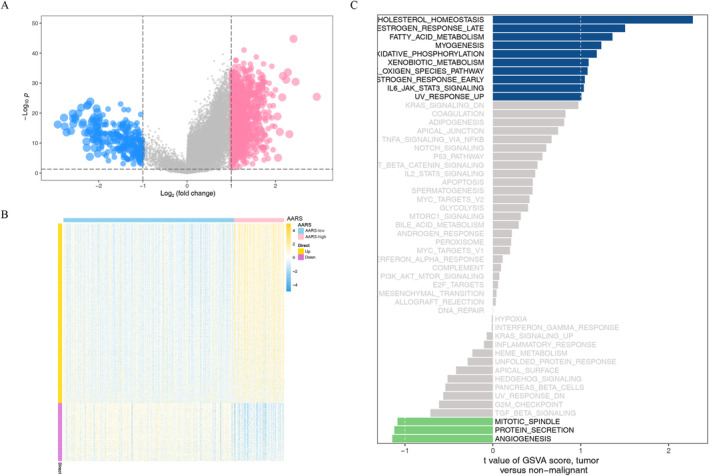
Comprehensive pathway enrichment analysis of the AARS score subgroups in TCGA‐LIHC cohort. (A) The volcano plot of DEGs between the AARS‐high and AARS‐low groups. (B) The heatmap of DEGs between the AARS‐high and AARS‐low groups. (C) GSVA analysis of the AARS‐high and AARS‐low groups.

We screened out 1150 DEGs in the TCGA‐LIHC cohort based on the AARS‐high and AARS‐low groups (Figure 6A). The proportion of upregulated DEGs in the AARS‐high group was higher than that of downregulated DEGs (Figure 6B). To understand the differences in biological functions and pathways among AARS‐high and AARS‐low groups, we conducted GSVA analysis, and the results demonstrated that the variation is concentrated in pathways of cholesterol metabolism, estrogen response late, fatty acid metabolism, and myogenesis metabolism (Figure 6C).

### 3.5 Evaluation of Immune Characteristics in AARS Subgroups

**FIGURE 7:**
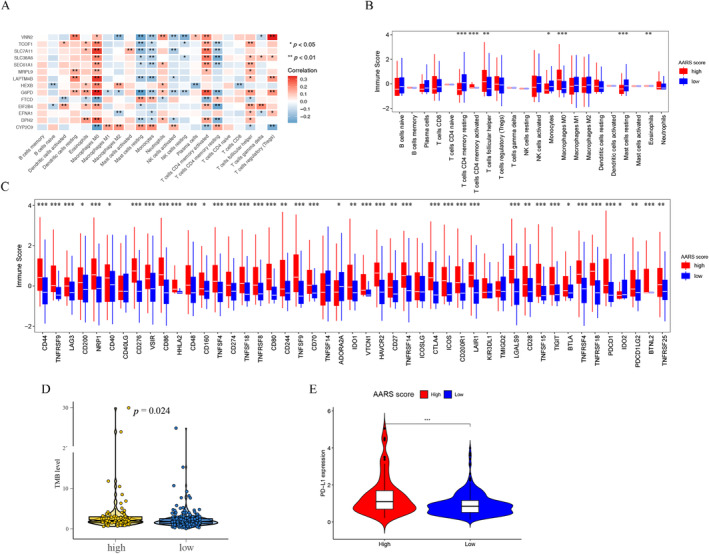
Immune characteristics of the AARS‐high and AARS‐low groups in the TCGA‐LIHC cohort. (A) The correlation between 22 types of immune cells and 14 amino acid‐related signatures. (B) Infiltration abundance of 22 types of immune cells in AARS‐high and AARS‐low groups. (C) Expression levels of 47 immune checkpoints in AARS‐high and AARS‐low groups. (D) TMB levels of the AARS‐high and AARS‐low groups. (E) PD‐L1 expression levels in the AARS‐high and AARS‐low groups.

The correlation between 14 amino acid‐related DEGs and 22 kinds of immune cells in the TCGA‐LIHC cohort was shown in Figure 7A. To fully understand the composition of the tumor microenvironment and potential ICI therapy response in the AARS score, we conducted a comprehensive analysis of immune checkpoints and TMB analysis. Figure 7B shows the relationship between the 22 kinds of immune cells and the two subgroups. In the AARS‐high group, immune scores of T cells follicular helper and macrophages M0 were higher than those of the AARS‐low group, while immune scores of T cell CD4 memory resting, monocytes, and mast cells resting were lower. The associations between 47 immune checkpoints and the AARS subgroups were visualized in Figure 7C. The immune scores of most immune checkpoints in the AARS‐high group were higher than those in the AARS‐low group. Since the expression of PD‐L1 and TMB was critical predictive markers of ICI and is widely used in clinics, we compared the two markers between the AARS subgroups. We found that the group AARS‐high had higher PD‐L1 expression and TMB level than the AARS‐low group (Figure 7D,E). The higher immune checkpoints, PD‐L1 expression, and TMB in the AARS‐high group indicated that the AARS‐high group might have a better response to ICI therapy.

### 3.6 Construction of Prognostic Nomogram

**FIGURE 8:**
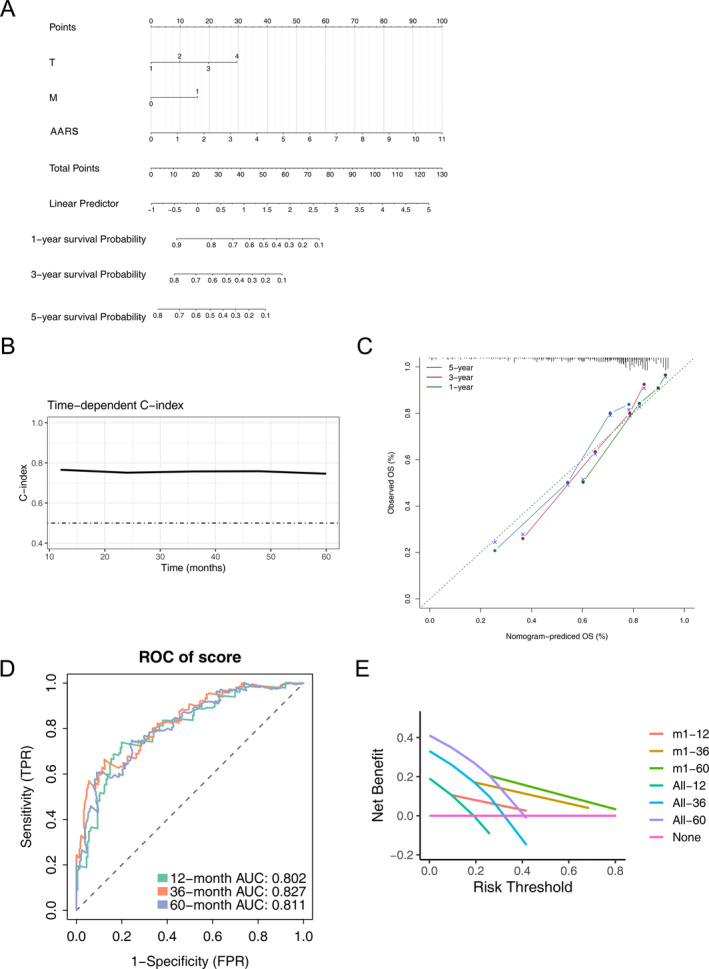
Nomogram of the AARS score and other clinical features for overall survival. (A) Nomogram for predicting 1‐, 3‐, and 5‐year OS in HCC patients. (B) Calibration curve of 1‐, 3‐ and 5‐year survival between the nomogram and the ideal model.

We incorporated the pathological stage, T stage, M stage, and the AARS to establish a predictive nomogram model for predicting the 1‐, 3‐ and 5‐year OS of HCC patients (Figure 8A). The calibration plot, used to analyze the nomogram prediction accuracy of OS in HCC, displayed that the nomogram had good prediction power (Figure 8B).

## 4 Discussion

In our study, we systematically investigated the characteristics of genes involved in amino acid‐related signatures in HCC tumor tissues and their relationship with the prognosis of HCC. A prognostic model incorporating 14 amino acid‐related genes was constructed and validated in an external cohort. We then constructed a clinical prediction model to achieve individualized survival prediction in patients with HCC.

In recent years, people have expanded the understanding of the risk factors and molecular characteristics of HCC with the development of bioinformatics. A growing body of research have reported that biomarkers based on energy metabolism could predict the prognosis of HCC. Chen et al. [25] discerned two distinct HCC subtypes with varying prognoses based on the metabolite–protein interactions network [25]. Hu et al. [26] identified and validated five genes associated with lipid metabolism, serving as prognostic indicators for OS in HCC [26]. He et al. [27] identified six fatty acid metabolic‐related genes that predict HCC prognosis [27]. Deng et al. [28] developed a glycolysis‐associated multiomics prognostic model (GMPM) that exhibited prognostic value in HCC [28]. However, there are still deficiencies in the research of amino acid metabolic reprogramming.

In our study, the AARS categorized patients with HCC into AARS‐high and AARS‐low groups. From K–M curve, it was observed that the patients in the AARS‐high group had a significantly shorter OS time than those in the AARS‐low group. The ROC curve substantiated the high accuracy of prognostic features predicting the survival of HCC patients. Univariate and multivariate Cox analyses indicated that AARS was an independent prognostic factor. Considering the heterogeneity of clinical characteristics among HCC patients and their impact on prognosis, we analyzed the predictive power of the AARS in different clinical groups from multiple perspectives in the training cohort. The results showed that in all clinical parameters, except G4 and N1, patients in the AARS‐high group presented shorter OS than those in the AARS‐low group. These results demonstrate the potential of AARS as a valuable tool for individualized prognostic assessments of HCC patients in diverse clinical stage.

We also constructed a predictive nomogram model for the training group, including pathological stage, T stage, M stage, and AARS. The practicability of the nomogram model was evaluated through a calibration chart. The results showed that it had good predictive and differential ability in monitoring OS of HCC patients.

We obtained 1150 differential genes by differential analysis between AARS‐high and AARS‐low groups. The expression of differential genes in the AARS‐high group was more active than that in the AARS‐low group. In the GSVA analysis, the variation was concentrated in the pathway associated with metabolism and biosynthesis, with a particular emphasis on cholesterol metabolism. Cholesterol, a lipid component essential for the formation of cell membranes and lipid rafts, plays a pivotal role in supporting the rapid growth of tumor cells. HCC features a specific cholesterol metabolic pathway involving the esterification of excess oxysterols induced by cholesterol acyltransferase 2 (ACAT 2). This pathway protects HCC cells from cytotoxicity induced by excessive oxidation sterol [29]. Recent research has shown that cholesterol induces the increase of taurocholic acid (TCA) and the decrease of 3‐indolepropionic acid (IPA), the metabolite of microbial tryptophan, through gut bacterial metabolites alteration, thus promoting the transition of nonalcoholic fatty liver disease to HCC [30]. Notably, FTCD, one of 14 amino acid‐related DEGs, is a liver‐specific enzyme in folate metabolism. The loss of FTCD has been demonstrated to exert an impact on the fatty acids and cholesterol metabolism during the development of liver cancer [31].

Considering the pivotal role of immunotherapy in HCC, it is essential to research the potential immune‐related mechanisms of prognostic markers in HCC. We found that 14 amino acid‐related DEGs were significantly correlated with immune cell infiltration. We further analyzed immune checkpoint, TMB, and PD‐L1 expression in the AARS subgroups. In the AARS‐high group, we observed that infiltration of T cell CD4 memory resting, monocytes, and mast cells resting was downregulated, while infiltration of T cells follicular helper and macrophages M0 was upregulated. Furthermore, we verified that the AARS‐high group had higher scores of immune checkpoints, TMB, and PD‐L1 expression than the AARS‐low group. These data indicate that for HCC patients treated with ICI, the AARS‐high group is more likely to experience treatment benefits.

We determined the AARS with strong prognostic value for HCC by Cox regression and Lasso method. Among the 14 variables, the biological significance of some genes has been reported in previous studies. FTCD is downregulated and involved in the occurrence of HCC [31, 32, 33]. MRPL9, a nuclear gene encoding protein component in mitochondrial ribosomes, is highly expressed in liver cancer and associated with the proliferation and migration of HCC [34]. EFNA1, a ligand of EphA2 receptor tyrosine kinase, is overexpressed in AFP‐producing HCC and can induce the expression of genes associated with tumor cell growth, angiogenesis, invasion, and metastasis, leading to poor prognosis in HCC patients with AFP [35]. G6PD, as a key enzyme in the pentose phosphate pathway, promotes the occurrence, migration, and invasion, as well as inhibited ferroptosis of HCC, which is correlated with poor prognosis and invasive clinicopathological features of HCC [36, 37]. Laptm4b‐35 encoded by LAPTM4B is overexpressed in more than 71% of HCC and can promote the proliferation, migration, and invasion of HCC [38]. The upregulation of SEC61A1 promotes HCC cell proliferation, migration, and stemness [39]. SLC38A6 plays an important role in glutamine metabolism, and its overexpression can promote HCC cell viability, colony formation, cell cycle progression, glutamine metabolism, and mitochondrial respiration [40]. SLC7A11, a member of the amino acid transporter SLC7 family, is induced by IL‐1β to upregulate PD‐L1 and CSF1 through αKG/HIF1α axis, leading to infiltration of tumor‐associated macrophages and myeloid‐derived suppressor cells and promoting metastasis of HCC [41]. Abnormally elevated TCOF1 coordinates oncogenic activation, rDNA transcription, and immune infiltration to promote HCC development [42]. VNN2, as a pantetheine hydrolase, is essential for pantothenic acid and coenzyme A biosynthesis. The upregulation of VNN2 can inhibit the proliferation, migration, and invasion of human osteosarcoma cells and induce their apoptosis [43]. The function of CYP2C9, DPH2, EIF2B4, and HEXB on cancer remains unclear, and their biological effects and clinical significance wait for further research in HCC.

There are some limitations and deficiencies existing in this study. First, the establishment and verification of prognostic signatures are based on retrospective analysis of public databases, introducing a degree of bias. The reliability and accuracy of this model need to be confirmed through further validation in prospective studies. Additionally, the correlation between some genes involved in this model and HCC remains unclear and needs in‐depth investigation.

## 5 Conclusion

Our study defines a novel prognosis model consisting of 14 amino acid‐related genes for HCC. This model was verified to independently predict the OS of HCC in both training and validation cohorts. In addition, our study provides effective prediction for stratification and personalized treatment of HCC, paving the way for more tailored prognostic assessments in the clinical setting. However, the underlying molecular mechanisms of these 14 genes in HCC remain to be further explored.

## Author Contributions

**Shuyi Wang:** methodology (equal), formal analysis (equal), visualization (equal), writing – original draft preparation (equal). **Hong Huang:** methodology (equal), formal analysis (equal), writing – original draft preparation (equal). **Xingwang Hu:** writing – review and editing (equal), supervision (equal). **Meifang Xiao:** writing – review and editing (equal), supervision (equal). **Kaili Yang:** writing – review and editing (equal). **Haiyan Bu:** writing – review and editing (equal). **Yupeng Jiang:** conceptualization (equal), methodology (equal), writing – review and editing (equal), project administration (equal), supervision (equal). **Zebing Huang:** conceptualization (equal), methodology (equal), writing – review and editing (equal), project administration (equal), supervision (equal).

## Ethics Statement

The authors have nothing to report.

## Consent

The authors have nothing to report.

## Conflicts of Interest

The authors declare no conflicts of interest.

## Supporting information


Table S1.

## Data Availability

The datasets generated and/or analyzed during the current study are available in The Cancer Genome Atlas (TCGA, https://portal.gdc.cancer.gov/) and the Gene Expression Omnibus (GEO, https://www.ncbi.nlm.nih.gov/geo/).
