# Supplementary material for: A Novel Amino Acid‐Related Gene Signature Predicts Overall Survival in Patients With Hepatocellular Carcinoma
Source: Cancer Rep (Hoboken). 2024 Jul 23;7(7):e2131. doi: 10.1002/cnr2.2131 (PMC11264112; doi:10.1002/cnr2.2131)
Supplement: Supplementary file 1 — Table S1. [file CNR2-7-e2131-s001.docx]

**Supplement Table 1.** Amino acid-related genes obtained from the Molecular Signatures Database

| Genes | Gene sets |
| --- | --- |
| AARS1 | amino acid and derivative metabolic process |
| ADI1 | amino acid and derivative metabolic process |
| AIMP1 | amino acid and derivative metabolic process |
| ALDH18A1 | amino acid and derivative metabolic process |
| ALDH4A1 | amino acid and derivative metabolic process |
| ALDH5A1 | amino acid and derivative metabolic process |
| ALDH6A1 | amino acid and derivative metabolic process |
| AMT | amino acid and derivative metabolic process |
| ARG1 | amino acid and derivative metabolic process |
| ASL | amino acid and derivative metabolic process |
| ASMTL | amino acid and derivative metabolic process |
| ASPA | amino acid and derivative metabolic process |
| ASRGL1 | amino acid and derivative metabolic process |
| ATF4 | amino acid and derivative metabolic process |
| BAAT | amino acid and derivative metabolic process |
| BBOX1 | amino acid and derivative metabolic process |
| BCAT1 | amino acid and derivative metabolic process |
| BCKDHA | amino acid and derivative metabolic process |
| BCKDHB | amino acid and derivative metabolic process |
| BCKDK | amino acid and derivative metabolic process |
| BPHL | amino acid and derivative metabolic process |
| CDO1 | amino acid and derivative metabolic process |
| COLQ | amino acid and derivative metabolic process |
| DARS1 | amino acid and derivative metabolic process |
| DCT | amino acid and derivative metabolic process |
| DDAH1 | amino acid and derivative metabolic process |
| DDAH2 | amino acid and derivative metabolic process |
| DDO | amino acid and derivative metabolic process |
| DHPS | amino acid and derivative metabolic process |
| DIO1 | amino acid and derivative metabolic process |
| DIO2 | amino acid and derivative metabolic process |
| ETNK1 | amino acid and derivative metabolic process |
| FAH | amino acid and derivative metabolic process |
| FARS2 | amino acid and derivative metabolic process |
| FPGS | amino acid and derivative metabolic process |
| GAD1 | amino acid and derivative metabolic process |
| GAD2 | amino acid and derivative metabolic process |
| GAMT | amino acid and derivative metabolic process |
| GATM | amino acid and derivative metabolic process |
| GCLC | amino acid and derivative metabolic process |
| GCLM | amino acid and derivative metabolic process |
| GCSH | amino acid and derivative metabolic process |
| GGT1 | amino acid and derivative metabolic process |
| GGT5 | amino acid and derivative metabolic process |
| GLDC | amino acid and derivative metabolic process |
| GLS2 | amino acid and derivative metabolic process |
| GLUD1 | amino acid and derivative metabolic process |
| GLUD2 | amino acid and derivative metabolic process |
| GOT1 | amino acid and derivative metabolic process |
| GOT2 | amino acid and derivative metabolic process |
| GSS | amino acid and derivative metabolic process |
| GSTZ1 | amino acid and derivative metabolic process |
| HDC | amino acid and derivative metabolic process |
| HGD | amino acid and derivative metabolic process |
| HPD | amino acid and derivative metabolic process |
| HPRT1 | amino acid and derivative metabolic process |
| IDO1 | amino acid and derivative metabolic process |
| KARS1 | amino acid and derivative metabolic process |
| KYAT1 | amino acid and derivative metabolic process |
| MARS2 | amino acid and derivative metabolic process |
| MAT1A | amino acid and derivative metabolic process |
| MAT2B | amino acid and derivative metabolic process |
| MCCC2 | amino acid and derivative metabolic process |
| MSRA | amino acid and derivative metabolic process |
| MTHFR | amino acid and derivative metabolic process |
| NFS1 | amino acid and derivative metabolic process |
| OAZ1 | amino acid and derivative metabolic process |
| OAZ2 | amino acid and derivative metabolic process |
| OTUB2 | amino acid and derivative metabolic process |
| P4HB | amino acid and derivative metabolic process |
| PAH | amino acid and derivative metabolic process |
| PEPD | amino acid and derivative metabolic process |
| PLOD1 | amino acid and derivative metabolic process |
| PRG3 | amino acid and derivative metabolic process |
| PTS | amino acid and derivative metabolic process |
| PYCR1 | amino acid and derivative metabolic process |
| QDPR | amino acid and derivative metabolic process |
| RARS1 | amino acid and derivative metabolic process |
| SARS2 | amino acid and derivative metabolic process |
| SCLY | amino acid and derivative metabolic process |
| SDS | amino acid and derivative metabolic process |
| SLC25A15 | amino acid and derivative metabolic process |
| SLC3A1 | amino acid and derivative metabolic process |
| SLC5A7 | amino acid and derivative metabolic process |
| SLC6A14 | amino acid and derivative metabolic process |
| SLC6A6 | amino acid and derivative metabolic process |
| SLC7A2 | amino acid and derivative metabolic process |
| SLC7A4 | amino acid and derivative metabolic process |
| SLC7A5 | amino acid and derivative metabolic process |
| SLC7A6 | amino acid and derivative metabolic process |
| SLC7A7 | amino acid and derivative metabolic process |
| SLC7A8 | amino acid and derivative metabolic process |
| SLC7A9 | amino acid and derivative metabolic process |
| SMS | amino acid and derivative metabolic process |
| SNCAIP | amino acid and derivative metabolic process |
| SULT1B1 | amino acid and derivative metabolic process |
| TGFB2 | amino acid and derivative metabolic process |
| TYR | amino acid and derivative metabolic process |
| WARS1 | amino acid and derivative metabolic process |
| YARS1 | amino acid and derivative metabolic process |
| YOD1 | amino acid and derivative metabolic process |
| AANAT | amide biosynthetic process |
| AARS2 | amide biosynthetic process |
| AARSD1 | amide biosynthetic process |
| AASDH | amide biosynthetic process |
| ABCA8 | amide biosynthetic process |
| ABCE1 | amide biosynthetic process |
| ABCF1 | amide biosynthetic process |
| ABTB1 | amide biosynthetic process |
| ACACA | amide biosynthetic process |
| ACACB | amide biosynthetic process |
| ACAT1 | amide biosynthetic process |
| ACLY | amide biosynthetic process |
| ACO1 | amide biosynthetic process |
| ACSBG1 | amide biosynthetic process |
| ACSBG2 | amide biosynthetic process |
| ACSF3 | amide biosynthetic process |
| ACSL1 | amide biosynthetic process |
| ACSL3 | amide biosynthetic process |
| ACSL4 | amide biosynthetic process |
| ACSL5 | amide biosynthetic process |
| ACSL6 | amide biosynthetic process |
| ACSS1 | amide biosynthetic process |
| ACSS2 | amide biosynthetic process |
| AGK | amide biosynthetic process |
| AGO1 | amide biosynthetic process |
| AGO2 | amide biosynthetic process |
| AGO3 | amide biosynthetic process |
| AGO4 | amide biosynthetic process |
| AIMP2 | amide biosynthetic process |
| AIRE | amide biosynthetic process |
| AKT1 | amide biosynthetic process |
| AKT2 | amide biosynthetic process |
| ALKBH1 | amide biosynthetic process |
| ALOX12B | amide biosynthetic process |
| ALOXE3 | amide biosynthetic process |
| ANG | amide biosynthetic process |
| APEH | amide biosynthetic process |
| APP | amide biosynthetic process |
| ARG2 | amide biosynthetic process |
| ASAH1 | amide biosynthetic process |
| ASAH2 | amide biosynthetic process |
| ASCC2 | amide biosynthetic process |
| ASCC3 | amide biosynthetic process |
| ASMT | amide biosynthetic process |
| ASNS | amide biosynthetic process |
| ASS1 | amide biosynthetic process |
| ATXN2 | amide biosynthetic process |
| B3GALT1 | amide biosynthetic process |
| B3GALT2 | amide biosynthetic process |
| B3GALT4 | amide biosynthetic process |
| B4GALNT1 | amide biosynthetic process |
| B4GALT3 | amide biosynthetic process |
| B4GALT4 | amide biosynthetic process |
| B4GALT5 | amide biosynthetic process |
| B4GALT6 | amide biosynthetic process |
| BACE1-AS | amide biosynthetic process |
| BANK1 | amide biosynthetic process |
| BARHL2 | amide biosynthetic process |
| BCL3 | amide biosynthetic process |
| BDH2 | amide biosynthetic process |
| BOLL | amide biosynthetic process |
| BTG2 | amide biosynthetic process |
| BZW1 | amide biosynthetic process |
| BZW2 | amide biosynthetic process |
| C1QBP | amide biosynthetic process |
| C8orf88 | amide biosynthetic process |
| CALR | amide biosynthetic process |
| CAPRIN1 | amide biosynthetic process |
| CAPRIN2 | amide biosynthetic process |
| CARNS1 | amide biosynthetic process |
| CARS1 | amide biosynthetic process |
| CARS2 | amide biosynthetic process |
| CASC3 | amide biosynthetic process |
| CCL5 | amide biosynthetic process |
| CCN1 | amide biosynthetic process |
| CD28 | amide biosynthetic process |
| CDC123 | amide biosynthetic process |
| CDK5RAP1 | amide biosynthetic process |
| CDKAL1 | amide biosynthetic process |
| CEBPA | amide biosynthetic process |
| CELF1 | amide biosynthetic process |
| CELF4 | amide biosynthetic process |
| CERS1 | amide biosynthetic process |
| CERS2 | amide biosynthetic process |
| CERS3 | amide biosynthetic process |
| CERS4 | amide biosynthetic process |
| CERS5 | amide biosynthetic process |
| CERS6 | amide biosynthetic process |
| CHAC1 | amide biosynthetic process |
| CHAC2 | amide biosynthetic process |
| CIRBP | amide biosynthetic process |
| CLN8 | amide biosynthetic process |
| CNBP | amide biosynthetic process |
| CNOT1 | amide biosynthetic process |
| CNOT10 | amide biosynthetic process |
| CNOT11 | amide biosynthetic process |
| CNOT2 | amide biosynthetic process |
| CNOT3 | amide biosynthetic process |
| CNOT6 | amide biosynthetic process |
| CNOT6L | amide biosynthetic process |
| CNOT7 | amide biosynthetic process |
| CNOT8 | amide biosynthetic process |
| CNOT9 | amide biosynthetic process |
| COA3 | amide biosynthetic process |
| COPS5 | amide biosynthetic process |
| CPEB1 | amide biosynthetic process |
| CPEB2 | amide biosynthetic process |
| CPEB3 | amide biosynthetic process |
| CPEB4 | amide biosynthetic process |
| CPS1 | amide biosynthetic process |
| CSDE1 | amide biosynthetic process |
| CTIF | amide biosynthetic process |
| CYFIP1 | amide biosynthetic process |
| CYP1B1 | amide biosynthetic process |
| CYP4F22 | amide biosynthetic process |
| DALRD3 | amide biosynthetic process |
| DAPK1 | amide biosynthetic process |
| DAPK3 | amide biosynthetic process |
| DARS2 | amide biosynthetic process |
| DAZ1 | amide biosynthetic process |
| DAZ2 | amide biosynthetic process |
| DAZ3 | amide biosynthetic process |
| DAZ4 | amide biosynthetic process |
| DAZL | amide biosynthetic process |
| DCP1A | amide biosynthetic process |
| DCP1B | amide biosynthetic process |
| DCP2 | amide biosynthetic process |
| DCPS | amide biosynthetic process |
| DDX1 | amide biosynthetic process |
| DDX25 | amide biosynthetic process |
| DDX3X | amide biosynthetic process |
| DDX6 | amide biosynthetic process |
| DEGS1 | amide biosynthetic process |
| DEGS2 | amide biosynthetic process |
| DENR | amide biosynthetic process |
| DHFR | amide biosynthetic process |
| DHFRP1 | amide biosynthetic process |
| DHX29 | amide biosynthetic process |
| DHX33 | amide biosynthetic process |
| DHX36 | amide biosynthetic process |
| DHX9 | amide biosynthetic process |
| DIP2A | amide biosynthetic process |
| DIS3 | amide biosynthetic process |
| DIS3L2 | amide biosynthetic process |
| DLAT | amide biosynthetic process |
| DLD | amide biosynthetic process |
| DMD | amide biosynthetic process |
| DNAJC1 | amide biosynthetic process |
| DNAJC24 | amide biosynthetic process |
| DNAJC3 | amide biosynthetic process |
| DND1 | amide biosynthetic process |
| DPH1 | amide biosynthetic process |
| DPH2 | amide biosynthetic process |
| DPH3 | amide biosynthetic process |
| DPH3P1 | amide biosynthetic process |
| DPH5 | amide biosynthetic process |
| DPH6 | amide biosynthetic process |
| DPH7 | amide biosynthetic process |
| DRG1 | amide biosynthetic process |
| DRG2 | amide biosynthetic process |
| EARS2 | amide biosynthetic process |
| EEF1A1 | amide biosynthetic process |
| EEF1A1P5 | amide biosynthetic process |
| EEF1A2 | amide biosynthetic process |
| EEF1B2 | amide biosynthetic process |
| EEF1D | amide biosynthetic process |
| EEF1E1 | amide biosynthetic process |
| EEF1G | amide biosynthetic process |
| EEF2 | amide biosynthetic process |
| EEF2K | amide biosynthetic process |
| EEFSEC | amide biosynthetic process |
| EFL1 | amide biosynthetic process |
| EIF1 | amide biosynthetic process |
| EIF1AD | amide biosynthetic process |
| EIF1AX | amide biosynthetic process |
| EIF1AY | amide biosynthetic process |
| EIF1B | amide biosynthetic process |
| EIF2A | amide biosynthetic process |
| EIF2AK1 | amide biosynthetic process |
| EIF2AK2 | amide biosynthetic process |
| EIF2AK3 | amide biosynthetic process |
| EIF2AK4 | amide biosynthetic process |
| EIF2B1 | amide biosynthetic process |
| EIF2B2 | amide biosynthetic process |
| EIF2B3 | amide biosynthetic process |
| EIF2B4 | amide biosynthetic process |
| EIF2B5 | amide biosynthetic process |
| EIF2D | amide biosynthetic process |
| EIF2S1 | amide biosynthetic process |
| EIF2S2 | amide biosynthetic process |
| EIF2S3 | amide biosynthetic process |
| EIF2S3B | amide biosynthetic process |
| EIF3A | amide biosynthetic process |
| EIF3B | amide biosynthetic process |
| EIF3C | amide biosynthetic process |
| EIF3CL | amide biosynthetic process |
| EIF3D | amide biosynthetic process |
| EIF3E | amide biosynthetic process |
| EIF3F | amide biosynthetic process |
| EIF3G | amide biosynthetic process |
| EIF3H | amide biosynthetic process |
| EIF3I | amide biosynthetic process |
| EIF3J | amide biosynthetic process |
| EIF3K | amide biosynthetic process |
| EIF3L | amide biosynthetic process |
| EIF3M | amide biosynthetic process |
| EIF4A1 | amide biosynthetic process |
| EIF4A2 | amide biosynthetic process |
| EIF4A3 | amide biosynthetic process |
| EIF4B | amide biosynthetic process |
| EIF4E | amide biosynthetic process |
| EIF4E1B | amide biosynthetic process |
| EIF4E2 | amide biosynthetic process |
| EIF4E3 | amide biosynthetic process |
| EIF4EBP1 | amide biosynthetic process |
| EIF4EBP2 | amide biosynthetic process |
| EIF4EBP3 | amide biosynthetic process |
| EIF4ENIF1 | amide biosynthetic process |
| EIF4G1 | amide biosynthetic process |
| EIF4G2 | amide biosynthetic process |
| EIF4G3 | amide biosynthetic process |
| EIF4H | amide biosynthetic process |
| EIF5 | amide biosynthetic process |
| EIF5A | amide biosynthetic process |
| EIF5A2 | amide biosynthetic process |
| EIF5AL1 | amide biosynthetic process |
| EIF5B | amide biosynthetic process |
| EIF6 | amide biosynthetic process |
| ELANE | amide biosynthetic process |
| ELAVL1 | amide biosynthetic process |
| ELAVL4 | amide biosynthetic process |
| ELOVL1 | amide biosynthetic process |
| ELOVL2 | amide biosynthetic process |
| ELOVL3 | amide biosynthetic process |
| ELOVL4 | amide biosynthetic process |
| ELOVL5 | amide biosynthetic process |
| ELOVL6 | amide biosynthetic process |
| ELOVL7 | amide biosynthetic process |
| ENC1 | amide biosynthetic process |
| ENPP7 | amide biosynthetic process |
| ENSG00000228549 | amide biosynthetic process |
| IARS2 | amide biosynthetic process |
| ERBB2 | amide biosynthetic process |
| ETF1 | amide biosynthetic process |
| EXOSC2 | amide biosynthetic process |
| EXOSC3 | amide biosynthetic process |
| EXOSC5 | amide biosynthetic process |
| EXOSC7 | amide biosynthetic process |
| EXOSC8 | amide biosynthetic process |
| EXOSC9 | amide biosynthetic process |
| FA2H | amide biosynthetic process |
| FARSA | amide biosynthetic process |
| FARSB | amide biosynthetic process |
| FASTKD2 | amide biosynthetic process |
| FASTKD3 | amide biosynthetic process |
| FMR1 | amide biosynthetic process |
| FOXO3 | amide biosynthetic process |
| FTO | amide biosynthetic process |
| FTSJ1 | amide biosynthetic process |
| FURIN | amide biosynthetic process |
| FXR1 | amide biosynthetic process |
| FXR2 | amide biosynthetic process |
| GAL3ST1 | amide biosynthetic process |
| GAPDH | amide biosynthetic process |
| GARS1 | amide biosynthetic process |
| GATB | amide biosynthetic process |
| GATC | amide biosynthetic process |
| GBA | amide biosynthetic process |
| GCDH | amide biosynthetic process |
| GCN1 | amide biosynthetic process |
| GEMIN5 | amide biosynthetic process |
| GFM1 | amide biosynthetic process |
| GFM2 | amide biosynthetic process |
| GGT2 | amide biosynthetic process |
| GGT3P | amide biosynthetic process |
| GGT6 | amide biosynthetic process |
| GGT7 | amide biosynthetic process |
| GGTA1 | amide biosynthetic process |
| GGTLC1 | amide biosynthetic process |
| GGTLC2 | amide biosynthetic process |
| GGTLC3 | amide biosynthetic process |
| GIGYF2 | amide biosynthetic process |
| GLE1 | amide biosynthetic process |
| GRB7 | amide biosynthetic process |
| GRM5 | amide biosynthetic process |
| GSPT1 | amide biosynthetic process |
| GSPT2 | amide biosynthetic process |
| GTPBP1 | amide biosynthetic process |
| GTPBP2 | amide biosynthetic process |
| GUF1 | amide biosynthetic process |
| GZMB | amide biosynthetic process |
| HABP4 | amide biosynthetic process |
| HACD1 | amide biosynthetic process |
| HACD2 | amide biosynthetic process |
| HAGH | amide biosynthetic process |
| HARS1 | amide biosynthetic process |
| HARS2 | amide biosynthetic process |
| HBS1L | amide biosynthetic process |
| HNRNPD | amide biosynthetic process |
| HNRNPU | amide biosynthetic process |
| HSD17B12 | amide biosynthetic process |
| HSPB1 | amide biosynthetic process |
| HTD2 | amide biosynthetic process |
| HYDIN2 | amide biosynthetic process |
| IARS1 | amide biosynthetic process |
| IARS2 | amide biosynthetic process |
| IGF2BP1 | amide biosynthetic process |
| IGF2BP2 | amide biosynthetic process |
| IGF2BP3 | amide biosynthetic process |
| IGFBP5 | amide biosynthetic process |
| IGHMBP2 | amide biosynthetic process |
| IL6 | amide biosynthetic process |
| ILF3 | amide biosynthetic process |
| IMPACT | amide biosynthetic process |
| INPP5E | amide biosynthetic process |
| IREB2 | amide biosynthetic process |
| ITGA2 | amide biosynthetic process |
| JMJD4 | amide biosynthetic process |
| KBTBD8 | amide biosynthetic process |
| KHDRBS1 | amide biosynthetic process |
| KHSRP | amide biosynthetic process |
| KLHL25 | amide biosynthetic process |
| KRT17 | amide biosynthetic process |
| LARP1 | amide biosynthetic process |
| LARP1B | amide biosynthetic process |
| LARP4 | amide biosynthetic process |
| LARP4B | amide biosynthetic process |
| LARP6 | amide biosynthetic process |
| LARS1 | amide biosynthetic process |
| LARS2 | amide biosynthetic process |
| LIN28A | amide biosynthetic process |
| LINC00324 | amide biosynthetic process |
| LINC01145 | amide biosynthetic process |
| LINC01783 | amide biosynthetic process |
| LINC02591 | amide biosynthetic process |
| LRPPRC | amide biosynthetic process |
| LRRC47 | amide biosynthetic process |
| LSM1 | amide biosynthetic process |
| LSM14A | amide biosynthetic process |
| LSM14B | amide biosynthetic process |
| LTN1 | amide biosynthetic process |
| LTO1 | amide biosynthetic process |
| MAGOH | amide biosynthetic process |
| MALSU1 | amide biosynthetic process |
| MAPKAPK5 | amide biosynthetic process |
| MARS1 | amide biosynthetic process |
| MCTS1 | amide biosynthetic process |
| METAP1 | amide biosynthetic process |
| METTL14 | amide biosynthetic process |
| METTL16 | amide biosynthetic process |
| METTL17 | amide biosynthetic process |
| METTL18 | amide biosynthetic process |
| METTL3 | amide biosynthetic process |
| METTL5 | amide biosynthetic process |
| MEX3D | amide biosynthetic process |
| MGST2 | amide biosynthetic process |
| MIF4GD | amide biosynthetic process |
| MIR1-1 | amide biosynthetic process |
| MIR100 | amide biosynthetic process |
| MIR101-1 | amide biosynthetic process |
| MIR103A1 | amide biosynthetic process |
| MIR106A | amide biosynthetic process |
| MIR106B | amide biosynthetic process |
| MIR107 | amide biosynthetic process |
| MIR10B | amide biosynthetic process |
| MIR125B1 | amide biosynthetic process |
| MIR1271 | amide biosynthetic process |
| MIR128-1 | amide biosynthetic process |
| MIR132 | amide biosynthetic process |
| MIR133B | amide biosynthetic process |
| MIR134 | amide biosynthetic process |
| MIR135B | amide biosynthetic process |
| MIR138-1 | amide biosynthetic process |
| MIR144 | amide biosynthetic process |
| MIR145 | amide biosynthetic process |
| MIR146A | amide biosynthetic process |
| MIR148A | amide biosynthetic process |
| MIR148B | amide biosynthetic process |
| MIR15A | amide biosynthetic process |
| MIR15B | amide biosynthetic process |
| MIR16-1 | amide biosynthetic process |
| MIR17 | amide biosynthetic process |
| MIR181A2 | amide biosynthetic process |
| MIR181B1 | amide biosynthetic process |
| MIR181C | amide biosynthetic process |
| MIR181D | amide biosynthetic process |
| MIR182 | amide biosynthetic process |
| MIR19B1 | amide biosynthetic process |
| MIR200B | amide biosynthetic process |
| MIR200C | amide biosynthetic process |
| MIR204 | amide biosynthetic process |
| MIR205 | amide biosynthetic process |
| MIR208A | amide biosynthetic process |
| MIR20A | amide biosynthetic process |
| MIR21 | amide biosynthetic process |
| MIR210 | amide biosynthetic process |
| MIR212 | amide biosynthetic process |
| MIR218-1 | amide biosynthetic process |
| MIR221 | amide biosynthetic process |
| MIR222 | amide biosynthetic process |
| MIR27A | amide biosynthetic process |
| MIR27B | amide biosynthetic process |
| MIR28 | amide biosynthetic process |
| MIR298 | amide biosynthetic process |
| MIR299 | amide biosynthetic process |
| MIR29A | amide biosynthetic process |
| MIR29B1 | amide biosynthetic process |
| MIR29C | amide biosynthetic process |
| MIR31 | amide biosynthetic process |
| MIR345 | amide biosynthetic process |
| MIR346 | amide biosynthetic process |
| MIR365A | amide biosynthetic process |
| MIR378A | amide biosynthetic process |
| MIR448 | amide biosynthetic process |
| MIR483 | amide biosynthetic process |
| MIR495 | amide biosynthetic process |
| MIR499A | amide biosynthetic process |
| MIR503 | amide biosynthetic process |
| MIR518B | amide biosynthetic process |
| MIR520B | amide biosynthetic process |
| MIR520C | amide biosynthetic process |
| MIR520E | amide biosynthetic process |
| MIR590 | amide biosynthetic process |
| MIR6086 | amide biosynthetic process |
| MIR659 | amide biosynthetic process |
| MIR877 | amide biosynthetic process |
| MIR9-1 | amide biosynthetic process |
| MIR92A1 | amide biosynthetic process |
| MIR939 | amide biosynthetic process |
| MIR96 | amide biosynthetic process |
| MIR98 | amide biosynthetic process |
| MIRLET7A1 | amide biosynthetic process |
| MIRLET7E | amide biosynthetic process |
| MIRLET7I | amide biosynthetic process |
| MKNK1 | amide biosynthetic process |
| MKNK2 | amide biosynthetic process |
| MLH1 | amide biosynthetic process |
| MLYCD | amide biosynthetic process |
| MOV10 | amide biosynthetic process |
| MPC2 | amide biosynthetic process |
| MPV17L2 | amide biosynthetic process |
| MRPL10 | amide biosynthetic process |
| MRPL11 | amide biosynthetic process |
| MRPL13 | amide biosynthetic process |
| MRPL16 | amide biosynthetic process |
| MRPL18 | amide biosynthetic process |
| MRPL2 | amide biosynthetic process |
| MRPL23 | amide biosynthetic process |
| MRPL24 | amide biosynthetic process |
| MRPL27 | amide biosynthetic process |
| MRPL28 | amide biosynthetic process |
| MRPL3 | amide biosynthetic process |
| MRPL32 | amide biosynthetic process |
| MRPL34 | amide biosynthetic process |
| MRPL35 | amide biosynthetic process |
| MRPL36 | amide biosynthetic process |
| MRPL37 | amide biosynthetic process |
| MRPL41 | amide biosynthetic process |
| MRPL42 | amide biosynthetic process |
| MRPL43 | amide biosynthetic process |
| MRPL44 | amide biosynthetic process |
| MRPL47 | amide biosynthetic process |
| MRPL51 | amide biosynthetic process |
| MRPL52 | amide biosynthetic process |
| MRPL55 | amide biosynthetic process |
| MRPL57 | amide biosynthetic process |
| MRPL58 | amide biosynthetic process |
| MRPL9 | amide biosynthetic process |
| MRPS11 | amide biosynthetic process |
| MRPS12 | amide biosynthetic process |
| MRPS14 | amide biosynthetic process |
| MRPS15 | amide biosynthetic process |
| MRPS16 | amide biosynthetic process |
| MRPS17 | amide biosynthetic process |
| MRPS18A | amide biosynthetic process |
| MRPS18B | amide biosynthetic process |
| MRPS18C | amide biosynthetic process |
| MRPS2 | amide biosynthetic process |
| MRPS21 | amide biosynthetic process |
| MRPS24 | amide biosynthetic process |
| MRPS27 | amide biosynthetic process |
| MRPS28 | amide biosynthetic process |
| MRPS33 | amide biosynthetic process |
| MRPS34 | amide biosynthetic process |
| MRPS5 | amide biosynthetic process |
| MRPS6 | amide biosynthetic process |
| MRPS7 | amide biosynthetic process |
| MRRF | amide biosynthetic process |
| MTFMT | amide biosynthetic process |
| MTG1 | amide biosynthetic process |
| MTG2 | amide biosynthetic process |
| MTIF2 | amide biosynthetic process |
| MTIF3 | amide biosynthetic process |
| MTOR | amide biosynthetic process |
| MTPN | amide biosynthetic process |
| MTRES1 | amide biosynthetic process |
| MTRF1 | amide biosynthetic process |
| MTRF1L | amide biosynthetic process |
| MTRFR | amide biosynthetic process |
| NAGS | amide biosynthetic process |
| NANOS1 | amide biosynthetic process |
| NANOS2 | amide biosynthetic process |
| NANOS3 | amide biosynthetic process |
| NANP | amide biosynthetic process |
| NARS1 | amide biosynthetic process |
| NARS2 | amide biosynthetic process |
| NAT10 | amide biosynthetic process |
| NCBP1 | amide biosynthetic process |
| NCBP2 | amide biosynthetic process |
| NCK1 | amide biosynthetic process |
| NCK2 | amide biosynthetic process |
| NCL | amide biosynthetic process |
| NDUFA7 | amide biosynthetic process |
| NEMF | amide biosynthetic process |
| NEURL1 | amide biosynthetic process |
| NFE2L2 | amide biosynthetic process |
| NGDN | amide biosynthetic process |
| NGRN | amide biosynthetic process |
| NIBAN1 | amide biosynthetic process |
| NOA1 | amide biosynthetic process |
| NOCT | amide biosynthetic process |
| NOD2 | amide biosynthetic process |
| NOLC1 | amide biosynthetic process |
| NPM1 | amide biosynthetic process |
| NSUN3 | amide biosynthetic process |
| NSUN5 | amide biosynthetic process |
| NT5C3B | amide biosynthetic process |
| OGFOD1 | amide biosynthetic process |
| ORMDL1 | amide biosynthetic process |
| ORMDL2 | amide biosynthetic process |
| ORMDL3 | amide biosynthetic process |
| OSBP | amide biosynthetic process |
| OTC | amide biosynthetic process |
| P2RX1 | amide biosynthetic process |
| P2RX7 | amide biosynthetic process |
| PA2G4 | amide biosynthetic process |
| PABPC1 | amide biosynthetic process |
| PABPC4 | amide biosynthetic process |
| PADI6 | amide biosynthetic process |
| PAIP1 | amide biosynthetic process |
| PAIP2 | amide biosynthetic process |
| PAIP2B | amide biosynthetic process |
| PAM | amide biosynthetic process |
| PAN2 | amide biosynthetic process |
| PAN3 | amide biosynthetic process |
| PARN | amide biosynthetic process |
| PARS2 | amide biosynthetic process |
| PASK | amide biosynthetic process |
| PATL1 | amide biosynthetic process |
| PATL2 | amide biosynthetic process |
| PCIF1 | amide biosynthetic process |
| PCSK1 | amide biosynthetic process |
| PCSK5 | amide biosynthetic process |
| PDE12 | amide biosynthetic process |
| PDF | amide biosynthetic process |
| PDHA1 | amide biosynthetic process |
| PDHA2 | amide biosynthetic process |
| PDHB | amide biosynthetic process |
| PDHX | amide biosynthetic process |
| PDK1 | amide biosynthetic process |
| PDK2 | amide biosynthetic process |
| PDK3 | amide biosynthetic process |
| PDK4 | amide biosynthetic process |
| PELO | amide biosynthetic process |
| PEMT | amide biosynthetic process |
| PER1 | amide biosynthetic process |
| PER2 | amide biosynthetic process |
| PINK1 | amide biosynthetic process |
| PIWIL1 | amide biosynthetic process |
| PIWIL2 | amide biosynthetic process |
| PIWIL3 | amide biosynthetic process |
| PIWIL4 | amide biosynthetic process |
| PKM | amide biosynthetic process |
| PKP3 | amide biosynthetic process |
| PLA2G6 | amide biosynthetic process |
| PLD1 | amide biosynthetic process |
| PLEKHN1 | amide biosynthetic process |
| PLXNB2 | amide biosynthetic process |
| PM20D1 | amide biosynthetic process |
| PNLDC1 | amide biosynthetic process |
| PNPLA1 | amide biosynthetic process |
| PNPT1 | amide biosynthetic process |
| POLDIP3 | amide biosynthetic process |
| POLR2D | amide biosynthetic process |
| POLR2G | amide biosynthetic process |
| PPCS | amide biosynthetic process |
| PPP1CA | amide biosynthetic process |
| PPP1R15A | amide biosynthetic process |
| PPP1R15B | amide biosynthetic process |
| PPT1 | amide biosynthetic process |
| PPT2 | amide biosynthetic process |
| PRKAA1 | amide biosynthetic process |
| PRKCD | amide biosynthetic process |
| PRKDC | amide biosynthetic process |
| PRR16 | amide biosynthetic process |
| PSTK | amide biosynthetic process |
| PTAFR | amide biosynthetic process |
| PTCD3 | amide biosynthetic process |
| PTK2B | amide biosynthetic process |
| PUM1 | amide biosynthetic process |
| PUM2 | amide biosynthetic process |
| PUM3 | amide biosynthetic process |
| PURA | amide biosynthetic process |
| PUS7 | amide biosynthetic process |
| PYM1 | amide biosynthetic process |
| QARS1 | amide biosynthetic process |
| QKI | amide biosynthetic process |
| QRSL1 | amide biosynthetic process |
| RACK1 | amide biosynthetic process |
| RARA | amide biosynthetic process |
| RARS2 | amide biosynthetic process |
| RBM24 | amide biosynthetic process |
| RBM3 | amide biosynthetic process |
| RBM4 | amide biosynthetic process |
| RBM4B | amide biosynthetic process |
| RBM8A | amide biosynthetic process |
| RC3H1 | amide biosynthetic process |
| RC3H2 | amide biosynthetic process |
| RCC1L | amide biosynthetic process |
| RGS2 | amide biosynthetic process |
| RHOA | amide biosynthetic process |
| RIDA | amide biosynthetic process |
| RMND1 | amide biosynthetic process |
| RNF139 | amide biosynthetic process |
| ROCK1 | amide biosynthetic process |
| ROCK2 | amide biosynthetic process |
| RPL10 | amide biosynthetic process |
| RPL10A | amide biosynthetic process |
| RPL10L | amide biosynthetic process |
| RPL11 | amide biosynthetic process |
| RPL12 | amide biosynthetic process |
| RPL13 | amide biosynthetic process |
| RPL13A | amide biosynthetic process |
| RPL13AP3 | amide biosynthetic process |
| RPL14 | amide biosynthetic process |
| RPL15 | amide biosynthetic process |
| RPL17 | amide biosynthetic process |
| RPL18 | amide biosynthetic process |
| RPL18A | amide biosynthetic process |
| RPL19 | amide biosynthetic process |
| RPL21 | amide biosynthetic process |
| RPL22 | amide biosynthetic process |
| RPL22L1 | amide biosynthetic process |
| RPL23 | amide biosynthetic process |
| RPL23A | amide biosynthetic process |
| RPL24 | amide biosynthetic process |
| RPL26 | amide biosynthetic process |
| RPL26L1 | amide biosynthetic process |
| RPL27 | amide biosynthetic process |
| RPL27A | amide biosynthetic process |
| RPL28 | amide biosynthetic process |
| RPL29 | amide biosynthetic process |
| RPL3 | amide biosynthetic process |
| RPL30 | amide biosynthetic process |
| RPL31 | amide biosynthetic process |
| RPL32 | amide biosynthetic process |
| RPL34 | amide biosynthetic process |
| RPL35 | amide biosynthetic process |
| RPL35A | amide biosynthetic process |
| RPL36 | amide biosynthetic process |
| RPL36A | amide biosynthetic process |
| RPL36AL | amide biosynthetic process |
| RPL37 | amide biosynthetic process |
| RPL37A | amide biosynthetic process |
| RPL37AP8 | amide biosynthetic process |
| RPL38 | amide biosynthetic process |
| RPL39 | amide biosynthetic process |
| RPL39L | amide biosynthetic process |
| RPL39P5 | amide biosynthetic process |
| RPL3L | amide biosynthetic process |
| RPL4 | amide biosynthetic process |
| RPL41 | amide biosynthetic process |
| RPL5 | amide biosynthetic process |
| RPL6 | amide biosynthetic process |
| RPL7 | amide biosynthetic process |
| RPL7A | amide biosynthetic process |
| RPL8 | amide biosynthetic process |
| RPL9 | amide biosynthetic process |
| RPLP0 | amide biosynthetic process |
| RPLP0P6 | amide biosynthetic process |
| RPLP1 | amide biosynthetic process |
| RPLP2 | amide biosynthetic process |
| RPS10 | amide biosynthetic process |
| RPS11 | amide biosynthetic process |
| RPS12 | amide biosynthetic process |
| RPS13 | amide biosynthetic process |
| RPS14 | amide biosynthetic process |
| RPS15 | amide biosynthetic process |
| RPS15A | amide biosynthetic process |
| RPS16 | amide biosynthetic process |
| RPS17 | amide biosynthetic process |
| RPS18 | amide biosynthetic process |
| RPS19 | amide biosynthetic process |
| RPS2 | amide biosynthetic process |
| RPS20 | amide biosynthetic process |
| RPS21 | amide biosynthetic process |
| RPS23 | amide biosynthetic process |
| RPS24 | amide biosynthetic process |
| RPS25 | amide biosynthetic process |
| RPS26 | amide biosynthetic process |
| RPS27 | amide biosynthetic process |
| RPS27A | amide biosynthetic process |
| RPS27L | amide biosynthetic process |
| RPS28 | amide biosynthetic process |
| RPS29 | amide biosynthetic process |
| RPS3 | amide biosynthetic process |
| RPS3A | amide biosynthetic process |
| RPS4X | amide biosynthetic process |
| RPS4Y1 | amide biosynthetic process |
| RPS4Y2 | amide biosynthetic process |
| RPS5 | amide biosynthetic process |
| RPS6 | amide biosynthetic process |
| RPS6KA1 | amide biosynthetic process |
| RPS6KA3 | amide biosynthetic process |
| RPS6KB1 | amide biosynthetic process |
| RPS6KB2 | amide biosynthetic process |
| RPS7 | amide biosynthetic process |
| RPS8 | amide biosynthetic process |
| RPS9 | amide biosynthetic process |
| RPSA | amide biosynthetic process |
| RPUSD3 | amide biosynthetic process |
| RPUSD4 | amide biosynthetic process |
| RRBP1 | amide biosynthetic process |
| RSL24D1 | amide biosynthetic process |
| RWDD1 | amide biosynthetic process |
| SAMD4A | amide biosynthetic process |
| SAMD4B | amide biosynthetic process |
| SAMD8 | amide biosynthetic process |
| SARNP | amide biosynthetic process |
| SARS1 | amide biosynthetic process |
| SECISBP2 | amide biosynthetic process |
| SECISBP2L | amide biosynthetic process |
| SELENOT | amide biosynthetic process |
| SEPSECS | amide biosynthetic process |
| SERP1 | amide biosynthetic process |
| SESN2 | amide biosynthetic process |
| SGMS1 | amide biosynthetic process |
| SGMS2 | amide biosynthetic process |
| SHFL | amide biosynthetic process |
| SHMT1 | amide biosynthetic process |
| SHMT2 | amide biosynthetic process |
| SIRT3 | amide biosynthetic process |
| SLBP | amide biosynthetic process |
| SLC1A1 | amide biosynthetic process |
| SLC1A2 | amide biosynthetic process |
| SLC25A1 | amide biosynthetic process |
| SLC25A2 | amide biosynthetic process |
| SLC7A11 | amide biosynthetic process |
| SMPD1 | amide biosynthetic process |
| SMPD2 | amide biosynthetic process |
| SMPD4 | amide biosynthetic process |
| SNCA | amide biosynthetic process |
| SOX4 | amide biosynthetic process |
| SPHK1 | amide biosynthetic process |
| SPHK2 | amide biosynthetic process |
| SPTLC1 | amide biosynthetic process |
| SPTLC2 | amide biosynthetic process |
| SPTLC3 | amide biosynthetic process |
| SPTSSA | amide biosynthetic process |
| SPTSSB | amide biosynthetic process |
| SRBD1 | amide biosynthetic process |
| SRP9 | amide biosynthetic process |
| ST3GAL1 | amide biosynthetic process |
| ST3GAL2 | amide biosynthetic process |
| ST3GAL3 | amide biosynthetic process |
| ST3GAL5 | amide biosynthetic process |
| ST6GALNAC3 | amide biosynthetic process |
| ST6GALNAC4 | amide biosynthetic process |
| ST6GALNAC5 | amide biosynthetic process |
| ST6GALNAC6 | amide biosynthetic process |
| ST8SIA2 | amide biosynthetic process |
| ST8SIA3 | amide biosynthetic process |
| ST8SIA4 | amide biosynthetic process |
| ST8SIA6 | amide biosynthetic process |
| STAT3 | amide biosynthetic process |
| SYNCRIP | amide biosynthetic process |
| TACO1 | amide biosynthetic process |
| TARBP2 | amide biosynthetic process |
| TARDBP | amide biosynthetic process |
| TARS1 | amide biosynthetic process |
| TARS2 | amide biosynthetic process |
| TARS3 | amide biosynthetic process |
| TCOF1 | amide biosynthetic process |
| TECR | amide biosynthetic process |
| TENT4A | amide biosynthetic process |
| TENT4B | amide biosynthetic process |
| TENT5B | amide biosynthetic process |
| THBS1 | amide biosynthetic process |
| TIA1 | amide biosynthetic process |
| TLCD3B | amide biosynthetic process |
| TMED2 | amide biosynthetic process |
| TNF | amide biosynthetic process |
| TNIP1 | amide biosynthetic process |
| TNRC6A | amide biosynthetic process |
| TNRC6B | amide biosynthetic process |
| TNRC6C | amide biosynthetic process |
| TOB1 | amide biosynthetic process |
| TPR | amide biosynthetic process |
| TRAP1 | amide biosynthetic process |
| TRIM71 | amide biosynthetic process |
| TRIP4 | amide biosynthetic process |
| TRMT10C | amide biosynthetic process |
| TRNAU1AP | amide biosynthetic process |
| TRUB2 | amide biosynthetic process |
| TSC1 | amide biosynthetic process |
| TSFM | amide biosynthetic process |
| TUFM | amide biosynthetic process |
| TUT4 | amide biosynthetic process |
| TUT7 | amide biosynthetic process |
| TYMS | amide biosynthetic process |
| UBA52 | amide biosynthetic process |
| UCN | amide biosynthetic process |
| UGCG | amide biosynthetic process |
| UGT8 | amide biosynthetic process |
| UHMK1 | amide biosynthetic process |
| UNK | amide biosynthetic process |
| UPF1 | amide biosynthetic process |
| UPF3A | amide biosynthetic process |
| UPF3B | amide biosynthetic process |
| UQCC2 | amide biosynthetic process |
| USP16 | amide biosynthetic process |
| VAPA | amide biosynthetic process |
| VARS1 | amide biosynthetic process |
| VARS2 | amide biosynthetic process |
| VIM | amide biosynthetic process |
| WARS2 | amide biosynthetic process |
| XRN1 | amide biosynthetic process |
| YARS2 | amide biosynthetic process |
| YBX1 | amide biosynthetic process |
| YBX2 | amide biosynthetic process |
| YBX3 | amide biosynthetic process |
| YTHDF1 | amide biosynthetic process |
| YTHDF2 | amide biosynthetic process |
| YTHDF3 | amide biosynthetic process |
| ZAR1 | amide biosynthetic process |
| ZAR1L | amide biosynthetic process |
| ZC3H12A | amide biosynthetic process |
| ZC3H12D | amide biosynthetic process |
| ZC3H15 | amide biosynthetic process |
| ZCCHC13 | amide biosynthetic process |
| ZCCHC4 | amide biosynthetic process |
| ZFP36 | amide biosynthetic process |
| ZFP36L1 | amide biosynthetic process |
| ZFP36L2 | amide biosynthetic process |
| ZNF385A | amide biosynthetic process |
| ZNF540 | amide biosynthetic process |
| ZNF598 | amide biosynthetic process |
| ZNF706 | amide biosynthetic process |
| ABAT | amine metabolic process |
| ACMSD | amine metabolic process |
| AFMID | amine metabolic process |
| AGMAT | amine metabolic process |
| ALDH2 | amine metabolic process |
| ALDH7A1 | amine metabolic process |
| AMD1 | amine metabolic process |
| AOC1 | amine metabolic process |
| AOC2 | amine metabolic process |
| AOC3 | amine metabolic process |
| ATCAY | amine metabolic process |
| ATP2B4 | amine metabolic process |
| ATP7A | amine metabolic process |
| AZIN1 | amine metabolic process |
| AZIN2 | amine metabolic process |
| BHMT | amine metabolic process |
| BLOC1S6 | amine metabolic process |
| CHDH | amine metabolic process |
| CHRNB2 | amine metabolic process |
| CLN3 | amine metabolic process |
| COMT | amine metabolic process |
| CYP1A1 | amine metabolic process |
| DAO | amine metabolic process |
| DBH | amine metabolic process |
| DDC | amine metabolic process |
| DMGDH | amine metabolic process |
| DRD1 | amine metabolic process |
| DRD2 | amine metabolic process |
| DRD3 | amine metabolic process |
| DRD4 | amine metabolic process |
| EDNRA | amine metabolic process |
| EPAS1 | amine metabolic process |
| GATA3 | amine metabolic process |
| GCH1 | amine metabolic process |
| GDE1 | amine metabolic process |
| GDPD1 | amine metabolic process |
| GDPD3 | amine metabolic process |
| GPR37 | amine metabolic process |
| GRIN2A | amine metabolic process |
| HAAO | amine metabolic process |
| HAND2 | amine metabolic process |
| HDAC10 | amine metabolic process |
| HDAC6 | amine metabolic process |
| HNMT | amine metabolic process |
| HTR1A | amine metabolic process |
| IDO2 | amine metabolic process |
| IL4I1 | amine metabolic process |
| INMT | amine metabolic process |
| INS | amine metabolic process |
| INSM1 | amine metabolic process |
| ITGAM | amine metabolic process |
| ITGB2 | amine metabolic process |
| KL | amine metabolic process |
| KMO | amine metabolic process |
| KYNU | amine metabolic process |
| LRTOMT | amine metabolic process |
| LY6E | amine metabolic process |
| MAOA | amine metabolic process |
| MAOB | amine metabolic process |
| MECP2 | amine metabolic process |
| MOXD1 | amine metabolic process |
| MOXD2P | amine metabolic process |
| NAAA | amine metabolic process |
| NAPEPLD | amine metabolic process |
| NNMT | amine metabolic process |
| NPR1 | amine metabolic process |
| NPY | amine metabolic process |
| NR1H4 | amine metabolic process |
| NR4A2 | amine metabolic process |
| OAZ3 | amine metabolic process |
| ODC1 | amine metabolic process |
| PAOX | amine metabolic process |
| PARK7 | amine metabolic process |
| PDE1B | amine metabolic process |
| PNKD | amine metabolic process |
| PNMT | amine metabolic process |
| PRKN | amine metabolic process |
| RNF180 | amine metabolic process |
| RTL4 | amine metabolic process |
| SAT1 | amine metabolic process |
| SAT2 | amine metabolic process |
| SIRT4 | amine metabolic process |
| SLC22A3 | amine metabolic process |
| SLC29A4 | amine metabolic process |
| SLC44A1 | amine metabolic process |
| SLC6A3 | amine metabolic process |
| SMOX | amine metabolic process |
| SNCB | amine metabolic process |
| SRM | amine metabolic process |
| SULT1A1 | amine metabolic process |
| SULT1A2 | amine metabolic process |
| SULT1A3 | amine metabolic process |
| SULT1A4 | amine metabolic process |
| SULT1C2 | amine metabolic process |
| TACR3 | amine metabolic process |
| TDO2 | amine metabolic process |
| TH | amine metabolic process |
| TRH | amine metabolic process |
| VCAM1 | amine metabolic process |
| VPS35 | amine metabolic process |
| ACE2 | amine transport |
| ADORA1 | amine transport |
| ADORA2A | amine transport |
| ADORA3 | amine transport |
| ADRA2A | amine transport |
| ADRA2B | amine transport |
| ADRA2C | amine transport |
| AGT | amine transport |
| AQP9 | amine transport |
| ARL6IP1 | amine transport |
| ARL6IP5 | amine transport |
| ATP1A2 | amine transport |
| AVP | amine transport |
| AVPR1A | amine transport |
| AVPR1B | amine transport |
| CARTPT | amine transport |
| CHGA | amine transport |
| CHRNA3 | amine transport |
| CHRNA4 | amine transport |
| CHRNA6 | amine transport |
| CLTRN | amine transport |
| CNR1 | amine transport |
| CRH | amine transport |
| CXCL12 | amine transport |
| DTNBP1 | amine transport |
| FFAR3 | amine transport |
| FGF20 | amine transport |
| GABBR1 | amine transport |
| GDNF | amine transport |
| GHSR | amine transport |
| GRK2 | amine transport |
| GRM2 | amine transport |
| GRM7 | amine transport |
| HRH3 | amine transport |
| HTR1B | amine transport |
| HTR2A | amine transport |
| ITGB1 | amine transport |
| KCNA2 | amine transport |
| KCNB1 | amine transport |
| LEP | amine transport |
| NPY5R | amine transport |
| NTSR1 | amine transport |
| OPRK1 | amine transport |
| OXT | amine transport |
| OXTR | amine transport |
| P2RY1 | amine transport |
| PSEN1 | amine transport |
| RAB3A | amine transport |
| RAB3B | amine transport |
| RAB3GAP1 | amine transport |
| RGS4 | amine transport |
| RHCG | amine transport |
| SLC12A2 | amine transport |
| SLC18A1 | amine transport |
| SLC18A2 | amine transport |
| SLC22A16 | amine transport |
| SLC22A2 | amine transport |
| SLC38A3 | amine transport |
| SLC43A1 | amine transport |
| SLC43A2 | amine transport |
| SLC6A1 | amine transport |
| SNCG | amine transport |
| STX1A | amine transport |
| STXBP1 | amine transport |
| SV2A | amine transport |
| SYT1 | amine transport |
| SYT10 | amine transport |
| SYT11 | amine transport |
| SYT12 | amine transport |
| SYT13 | amine transport |
| SYT15 | amine transport |
| SYT17 | amine transport |
| SYT2 | amine transport |
| SYT3 | amine transport |
| SYT4 | amine transport |
| SYT5 | amine transport |
| SYT6 | amine transport |
| SYT7 | amine transport |
| SYT8 | amine transport |
| SYT9 | amine transport |
| TACR2 | amine transport |
| TOR1A | amine transport |
| VIP | amine transport |
| GFAP | amino acid transmembrane transport |
| GRM1 | amino acid transmembrane transport |
| KCNJ10 | amino acid transmembrane transport |
| LRRC8A | amino acid transmembrane transport |
| LRRC8C | amino acid transmembrane transport |
| LRRC8D | amino acid transmembrane transport |
| LRRC8E | amino acid transmembrane transport |
| MFSD12 | amino acid transmembrane transport |
| PRAF2 | amino acid transmembrane transport |
| SFXN1 | amino acid transmembrane transport |
| SFXN2 | amino acid transmembrane transport |
| SFXN3 | amino acid transmembrane transport |
| SLC15A4 | amino acid transmembrane transport |
| SLC16A10 | amino acid transmembrane transport |
| SLC16A2 | amino acid transmembrane transport |
| SLC17A6 | amino acid transmembrane transport |
| SLC17A7 | amino acid transmembrane transport |
| SLC17A8 | amino acid transmembrane transport |
| SLC1A3 | amino acid transmembrane transport |
| SLC1A4 | amino acid transmembrane transport |
| SLC1A5 | amino acid transmembrane transport |
| SLC1A6 | amino acid transmembrane transport |
| SLC1A7 | amino acid transmembrane transport |
| SLC22A4 | amino acid transmembrane transport |
| SLC25A12 | amino acid transmembrane transport |
| SLC25A13 | amino acid transmembrane transport |
| SLC25A18 | amino acid transmembrane transport |
| SLC25A22 | amino acid transmembrane transport |
| SLC25A29 | amino acid transmembrane transport |
| SLC25A38 | amino acid transmembrane transport |
| SLC32A1 | amino acid transmembrane transport |
| SLC36A1 | amino acid transmembrane transport |
| SLC36A2 | amino acid transmembrane transport |
| SLC36A3 | amino acid transmembrane transport |
| SLC36A4 | amino acid transmembrane transport |
| SLC38A1 | amino acid transmembrane transport |
| SLC38A10 | amino acid transmembrane transport |
| SLC38A11 | amino acid transmembrane transport |
| SLC38A2 | amino acid transmembrane transport |
| SLC38A4 | amino acid transmembrane transport |
| SLC38A5 | amino acid transmembrane transport |
| SLC38A6 | amino acid transmembrane transport |
| SLC38A7 | amino acid transmembrane transport |
| SLC38A8 | amino acid transmembrane transport |
| SLC38A9 | amino acid transmembrane transport |
| SLC3A2 | amino acid transmembrane transport |
| SLC47A1 | amino acid transmembrane transport |
| SLC66A1 | amino acid transmembrane transport |
| SLC66A1L | amino acid transmembrane transport |
| SLC6A12 | amino acid transmembrane transport |
| SLC6A13 | amino acid transmembrane transport |
| SLC6A15 | amino acid transmembrane transport |
| SLC6A18 | amino acid transmembrane transport |
| SLC6A19 | amino acid transmembrane transport |
| SLC6A20 | amino acid transmembrane transport |
| SLC6A5 | amino acid transmembrane transport |
| SLC6A7 | amino acid transmembrane transport |
| SLC6A9 | amino acid transmembrane transport |
| SLC7A1 | amino acid transmembrane transport |
| SLC7A10 | amino acid transmembrane transport |
| SLC7A13 | amino acid transmembrane transport |
| SLC7A14 | amino acid transmembrane transport |
| SLC7A3 | amino acid transmembrane transport |
| SLC7A5P1 | amino acid transmembrane transport |
| SLC7A5P2 | amino acid transmembrane transport |
| TSPO2 | amino acid transmembrane transport |
| APBA1 | amino acid transport |
| CTNS | amino acid transport |
| GIPC1 | amino acid transport |
| GJA1 | amino acid transport |
| LLGL2 | amino acid transport |
| MYC | amino acid transport |
| NF1 | amino acid transport |
| NTRK2 | amino acid transport |
| OCA2 | amino acid transport |
| PDPN | amino acid transport |
| SERINC3 | amino acid transport |
| SERINC5 | amino acid transport |
| SFXN4 | amino acid transport |
| SFXN5 | amino acid transport |
| SH3BP4 | amino acid transport |
| SLC17A5 | amino acid transport |
| SLC25A44 | amino acid transport |
| SLC25A45 | amino acid transport |
| SLC25A47 | amino acid transport |
| SLC25A48 | amino acid transport |
| SLC6A11 | amino acid transport |
| SLC6A17 | amino acid transport |
| SLC9A3R1 | amino acid transport |
| TRPC4 | amino acid transport |
| TRPV1 | amino acid transport |
| XK | amino acid transport |
| AADAT | cellular amide metabolic process |
| AASDHPPT | cellular amide metabolic process |
| AASS | cellular amide metabolic process |
| ABCA12 | cellular amide metabolic process |
| ABCA2 | cellular amide metabolic process |
| ABCA7 | cellular amide metabolic process |
| ABCB9 | cellular amide metabolic process |
| ABCD1 | cellular amide metabolic process |
| ABCG1 | cellular amide metabolic process |
| ABHD4 | cellular amide metabolic process |
| ACE | cellular amide metabolic process |
| ACER1 | cellular amide metabolic process |
| ACER2 | cellular amide metabolic process |
| ACER3 | cellular amide metabolic process |
| ACOT1 | cellular amide metabolic process |
| ACOT11 | cellular amide metabolic process |
| ACOT12 | cellular amide metabolic process |
| ACOT2 | cellular amide metabolic process |
| ACOT4 | cellular amide metabolic process |
| ACOT6 | cellular amide metabolic process |
| ACOT7 | cellular amide metabolic process |
| ACOT8 | cellular amide metabolic process |
| ACOT9 | cellular amide metabolic process |
| ACSF2 | cellular amide metabolic process |
| ACSM1 | cellular amide metabolic process |
| ACSM2A | cellular amide metabolic process |
| ACSM2B | cellular amide metabolic process |
| ACSM3 | cellular amide metabolic process |
| ACSM4 | cellular amide metabolic process |
| ACSM5 | cellular amide metabolic process |
| ACSM6 | cellular amide metabolic process |
| ADA | cellular amide metabolic process |
| ADAMTS13 | cellular amide metabolic process |
| AEBP1 | cellular amide metabolic process |
| ALDH1L2 | cellular amide metabolic process |
| AMDHD1 | cellular amide metabolic process |
| AMDHD2 | cellular amide metabolic process |
| ANPEP | cellular amide metabolic process |
| APH1A | cellular amide metabolic process |
| APH1B | cellular amide metabolic process |
| APOE | cellular amide metabolic process |
| ASAH2B | cellular amide metabolic process |
| ATP6AP2 | cellular amide metabolic process |
| BACE1 | cellular amide metabolic process |
| BACE2 | cellular amide metabolic process |
| BCHE | cellular amide metabolic process |
| BECN1 | cellular amide metabolic process |
| BIN1 | cellular amide metabolic process |
| BTD | cellular amide metabolic process |
| CARNMT1 | cellular amide metabolic process |
| CASP3 | cellular amide metabolic process |
| CEL | cellular amide metabolic process |
| CERK | cellular amide metabolic process |
| CERT1 | cellular amide metabolic process |
| CHRNA7 | cellular amide metabolic process |
| CLIC1 | cellular amide metabolic process |
| CLIC2 | cellular amide metabolic process |
| CLIC3 | cellular amide metabolic process |
| CLIC5 | cellular amide metabolic process |
| CLN5 | cellular amide metabolic process |
| CLN6 | cellular amide metabolic process |
| CLU | cellular amide metabolic process |
| CMA1 | cellular amide metabolic process |
| CMAS | cellular amide metabolic process |
| CORIN | cellular amide metabolic process |
| CPA3 | cellular amide metabolic process |
| CPD | cellular amide metabolic process |
| CPE | cellular amide metabolic process |
| CPM | cellular amide metabolic process |
| CPN1 | cellular amide metabolic process |
| CPQ | cellular amide metabolic process |
| CPXM1 | cellular amide metabolic process |
| CPXM2 | cellular amide metabolic process |
| CPZ | cellular amide metabolic process |
| CTSG | cellular amide metabolic process |
| CTSH | cellular amide metabolic process |
| CTSL | cellular amide metabolic process |
| CTSZ | cellular amide metabolic process |
| CYP2C9 | cellular amide metabolic process |
| DGAT1 | cellular amide metabolic process |
| DGAT2 | cellular amide metabolic process |
| DHFR2 | cellular amide metabolic process |
| DISP1 | cellular amide metabolic process |
| DLST | cellular amide metabolic process |
| DNPEP | cellular amide metabolic process |
| DPEP1 | cellular amide metabolic process |
| DPP4 | cellular amide metabolic process |
| DYRK1A | cellular amide metabolic process |
| ECE1 | cellular amide metabolic process |
| ECE2 | cellular amide metabolic process |
| EFNA1 | cellular amide metabolic process |
| ENPEP | cellular amide metabolic process |
| ENSG00000205794 | cellular amide metabolic process |
| EPHA4 | cellular amide metabolic process |
| ERAP1 | cellular amide metabolic process |
| ERAP2 | cellular amide metabolic process |
| ERO1A | cellular amide metabolic process |
| ETHE1 | cellular amide metabolic process |
| FAR1 | cellular amide metabolic process |
| FAR2 | cellular amide metabolic process |
| FITM2 | cellular amide metabolic process |
| FOLR1 | cellular amide metabolic process |
| FTCD | cellular amide metabolic process |
| FUT3 | cellular amide metabolic process |
| FUT5 | cellular amide metabolic process |
| FUT6 | cellular amide metabolic process |
| FUT7 | cellular amide metabolic process |
| G6PD | cellular amide metabolic process |
| GALC | cellular amide metabolic process |
| GBA2 | cellular amide metabolic process |
| GBA3 | cellular amide metabolic process |
| GDA | cellular amide metabolic process |
| GDAP1 | cellular amide metabolic process |
| GDAP1L1 | cellular amide metabolic process |
| GGA3 | cellular amide metabolic process |
| GLA | cellular amide metabolic process |
| GLO1 | cellular amide metabolic process |
| GLRX2 | cellular amide metabolic process |
| GLYAT | cellular amide metabolic process |
| GM2A | cellular amide metabolic process |
| GNE | cellular amide metabolic process |
| GNPDA1 | cellular amide metabolic process |
| GNPDA2 | cellular amide metabolic process |
| GPAM | cellular amide metabolic process |
| GPAT4 | cellular amide metabolic process |
| GPX1 | cellular amide metabolic process |
| GPX4 | cellular amide metabolic process |
| GSAP | cellular amide metabolic process |
| GSK3A | cellular amide metabolic process |
| GSR | cellular amide metabolic process |
| GSTA1 | cellular amide metabolic process |
| GSTA2 | cellular amide metabolic process |
| GSTA3 | cellular amide metabolic process |
| GSTA4 | cellular amide metabolic process |
| GSTA5 | cellular amide metabolic process |
| GSTK1 | cellular amide metabolic process |
| GSTM1 | cellular amide metabolic process |
| GSTM2 | cellular amide metabolic process |
| GSTM3 | cellular amide metabolic process |
| GSTM4 | cellular amide metabolic process |
| GSTM5 | cellular amide metabolic process |
| GSTO1 | cellular amide metabolic process |
| GSTO2 | cellular amide metabolic process |
| GSTP1 | cellular amide metabolic process |
| GSTT1 | cellular amide metabolic process |
| GSTT2 | cellular amide metabolic process |
| GSTT2B | cellular amide metabolic process |
| GSTT4 | cellular amide metabolic process |
| HAL | cellular amide metabolic process |
| HAP1 | cellular amide metabolic process |
| HEXA | cellular amide metabolic process |
| HEXB | cellular amide metabolic process |
| HLCS | cellular amide metabolic process |
| HM13 | cellular amide metabolic process |
| HMGCS1 | cellular amide metabolic process |
| HMGCS2 | cellular amide metabolic process |
| HPGDS | cellular amide metabolic process |
| HSD17B4 | cellular amide metabolic process |
| HTRA2 | cellular amide metabolic process |
| IDE | cellular amide metabolic process |
| IDH1 | cellular amide metabolic process |
| IFNG | cellular amide metabolic process |
| IFNGR1 | cellular amide metabolic process |
| IGF1 | cellular amide metabolic process |
| ITGB8 | cellular amide metabolic process |
| LNPEP | cellular amide metabolic process |
| LRRTM3 | cellular amide metabolic process |
| LTA4H | cellular amide metabolic process |
| LVRN | cellular amide metabolic process |
| MBOAT4 | cellular amide metabolic process |
| MCCC1 | cellular amide metabolic process |
| MCEE | cellular amide metabolic process |
| MGAT3 | cellular amide metabolic process |
| MIPEP | cellular amide metabolic process |
| MIR153-1 | cellular amide metabolic process |
| MIR186 | cellular amide metabolic process |
| MIR206 | cellular amide metabolic process |
| MIR24-1 | cellular amide metabolic process |
| MIR339 | cellular amide metabolic process |
| MIR361 | cellular amide metabolic process |
| MIR455 | cellular amide metabolic process |
| MMACHC | cellular amide metabolic process |
| MME | cellular amide metabolic process |
| MTHFD2 | cellular amide metabolic process |
| MTHFD2L | cellular amide metabolic process |
| MTHFS | cellular amide metabolic process |
| MTRR | cellular amide metabolic process |
| MVD | cellular amide metabolic process |
| MVK | cellular amide metabolic process |
| NAALADL1 | cellular amide metabolic process |
| NAGA | cellular amide metabolic process |
| NAGK | cellular amide metabolic process |
| NAT8 | cellular amide metabolic process |
| NAT8B | cellular amide metabolic process |
| NCSTN | cellular amide metabolic process |
| NEU1 | cellular amide metabolic process |
| NEU2 | cellular amide metabolic process |
| NEU3 | cellular amide metabolic process |
| NEU4 | cellular amide metabolic process |
| NIT1 | cellular amide metabolic process |
| NLN | cellular amide metabolic process |
| NPEPPS | cellular amide metabolic process |
| NPEPPSP1 | cellular amide metabolic process |
| NPL | cellular amide metabolic process |
| NSMAF | cellular amide metabolic process |
| NT5C | cellular amide metabolic process |
| NT5C1A | cellular amide metabolic process |
| NT5C2 | cellular amide metabolic process |
| NUDT19 | cellular amide metabolic process |
| NUDT7 | cellular amide metabolic process |
| NUDT8 | cellular amide metabolic process |
| OGDH | cellular amide metabolic process |
| OPLAH | cellular amide metabolic process |
| OXSM | cellular amide metabolic process |
| PANK2 | cellular amide metabolic process |
| PARL | cellular amide metabolic process |
| PCSK1N | cellular amide metabolic process |
| PCSK2 | cellular amide metabolic process |
| PCSK4 | cellular amide metabolic process |
| PCSK6 | cellular amide metabolic process |
| PCSK7 | cellular amide metabolic process |
| PGAM1 | cellular amide metabolic process |
| PGLYRP2 | cellular amide metabolic process |
| PICALM | cellular amide metabolic process |
| PIN1 | cellular amide metabolic process |
| PIPOX | cellular amide metabolic process |
| PLA2G15 | cellular amide metabolic process |
| PLA2G4E | cellular amide metabolic process |
| PLA2G7 | cellular amide metabolic process |
| PLAAT1 | cellular amide metabolic process |
| PLAAT2 | cellular amide metabolic process |
| PLAAT3 | cellular amide metabolic process |
| PLAAT4 | cellular amide metabolic process |
| PLAAT5 | cellular amide metabolic process |
| PLPP1 | cellular amide metabolic process |
| PLPP2 | cellular amide metabolic process |
| PLPP3 | cellular amide metabolic process |
| PM20D2 | cellular amide metabolic process |
| PMVK | cellular amide metabolic process |
| PNP | cellular amide metabolic process |
| PPP2CA | cellular amide metabolic process |
| PPP2R1A | cellular amide metabolic process |
| PRNP | cellular amide metabolic process |
| PSEN2 | cellular amide metabolic process |
| PSENEN | cellular amide metabolic process |
| PTGES2 | cellular amide metabolic process |
| RELA | cellular amide metabolic process |
| REN | cellular amide metabolic process |
| RENBP | cellular amide metabolic process |
| RTN1 | cellular amide metabolic process |
| RTN2 | cellular amide metabolic process |
| RTN3 | cellular amide metabolic process |
| RTN4 | cellular amide metabolic process |
| SCG5 | cellular amide metabolic process |
| SEC11A | cellular amide metabolic process |
| SEC11B | cellular amide metabolic process |
| SEC11C | cellular amide metabolic process |
| SGPL1 | cellular amide metabolic process |
| SLC19A1 | cellular amide metabolic process |
| SLC25A32 | cellular amide metabolic process |
| SLC2A13 | cellular amide metabolic process |
| SLC30A5 | cellular amide metabolic process |
| SLC30A6 | cellular amide metabolic process |
| SLC30A8 | cellular amide metabolic process |
| SLC46A1 | cellular amide metabolic process |
| SMPD3 | cellular amide metabolic process |
| SMPDL3A | cellular amide metabolic process |
| SMPDL3B | cellular amide metabolic process |
| SOD1 | cellular amide metabolic process |
| SOD2 | cellular amide metabolic process |
| SORL1 | cellular amide metabolic process |
| SP1 | cellular amide metabolic process |
| SPCS1 | cellular amide metabolic process |
| SPCS2 | cellular amide metabolic process |
| SPCS3 | cellular amide metabolic process |
| SPON1 | cellular amide metabolic process |
| SPPL3 | cellular amide metabolic process |
| ST6GAL1 | cellular amide metabolic process |
| SUCLA2 | cellular amide metabolic process |
| SUCLG2 | cellular amide metabolic process |
| TAPBP | cellular amide metabolic process |
| THEM5 | cellular amide metabolic process |
| THOP1 | cellular amide metabolic process |
| TIGAR | cellular amide metabolic process |
| TM9SF2 | cellular amide metabolic process |
| TMED10 | cellular amide metabolic process |
| TP53 | cellular amide metabolic process |
| TPP1 | cellular amide metabolic process |
| TRHDE | cellular amide metabolic process |
| URAD | cellular amide metabolic process |
| UROC1 | cellular amide metabolic process |
| VNN1 | cellular amide metabolic process |
| VNN2 | cellular amide metabolic process |
| VNN3 | cellular amide metabolic process |
| XDH | cellular amide metabolic process |
| XPNPEP1 | cellular amide metabolic process |
| YIPF5 | cellular amide metabolic process |
| ACAD9 | amide binding |
| ACADL | amide binding |
| ACADVL | amide binding |
| ACBD3 | amide binding |
| ACBD4 | amide binding |
| ACBD5 | amide binding |
| ACBD6 | amide binding |
| ACBD7 | amide binding |
| ACHE | amide binding |
| ACVR1 | amide binding |
| ADCYAP1R1 | amide binding |
| ADNP | amide binding |
| ADRB2 | amide binding |
| AGER | amide binding |
| AP2B1 | amide binding |
| AP2M1 | amide binding |
| APBA2 | amide binding |
| APBA3 | amide binding |
| APBB1 | amide binding |
| APBB2 | amide binding |
| APBB3 | amide binding |
| APOA1 | amide binding |
| ATP1A3 | amide binding |
| AVPR2 | amide binding |
| BABAM2 | amide binding |
| BDKRB1 | amide binding |
| BRAP | amide binding |
| C1QA | amide binding |
| C2CD2L | amide binding |
| CABP1 | amide binding |
| CACNA1A | amide binding |
| CACNA1B | amide binding |
| CALCR | amide binding |
| CALCRL | amide binding |
| CAT | amide binding |
| CCKAR | amide binding |
| CCKBR | amide binding |
| CD14 | amide binding |
| CD1A | amide binding |
| CD1B | amide binding |
| CD1C | amide binding |
| CD1D | amide binding |
| CD1E | amide binding |
| CD209 | amide binding |
| CD300LF | amide binding |
| CD36 | amide binding |
| CD74 | amide binding |
| CEMIP | amide binding |
| CLEC4M | amide binding |
| CLIP3 | amide binding |
| CLSTN1 | amide binding |
| CLTA | amide binding |
| CLTB | amide binding |
| CMKLR1 | amide binding |
| CMKLR2 | amide binding |
| COL25A1 | amide binding |
| CPTP | amide binding |
| CRHBP | amide binding |
| CRHR1 | amide binding |
| CRHR2 | amide binding |
| CRIP1 | amide binding |
| CRYAB | amide binding |
| CST3 | amide binding |
| CWC27 | amide binding |
| DBI | amide binding |
| DHCR24 | amide binding |
| DLGAP3 | amide binding |
| EBI3 | amide binding |
| ECI2 | amide binding |
| EDNRB | amide binding |
| EPDR1 | amide binding |
| EPHB2 | amide binding |
| FASN | amide binding |
| FBXO2 | amide binding |
| FCGR2B | amide binding |
| FCGRT | amide binding |
| FKBP10 | amide binding |
| FKBP1A | amide binding |
| FKBP1B | amide binding |
| FKBP2 | amide binding |
| FKBP3 | amide binding |
| FKBP4 | amide binding |
| FKBP5 | amide binding |
| FKBP6 | amide binding |
| FKBP7 | amide binding |
| FOLH1 | amide binding |
| FOLR2 | amide binding |
| FOLR3 | amide binding |
| FPR2 | amide binding |
| FTCDNL1 | amide binding |
| FZD4 | amide binding |
| FZD5 | amide binding |
| FZD6 | amide binding |
| GALR1 | amide binding |
| GALR2 | amide binding |
| GALR3 | amide binding |
| GCGR | amide binding |
| GHR | amide binding |
| GHRHR | amide binding |
| GIPR | amide binding |
| GLP1R | amide binding |
| GLP2R | amide binding |
| GLTP | amide binding |
| GLTPD2 | amide binding |
| GNMT | amide binding |
| GNRHR | amide binding |
| GNRHR2 | amide binding |
| GPR149 | amide binding |
| GPR150 | amide binding |
| GPR22 | amide binding |
| GPR37L1 | amide binding |
| GRIA1 | amide binding |
| GRIA2 | amide binding |
| GRIA3 | amide binding |
| GRIA4 | amide binding |
| GRIN1 | amide binding |
| GRIN2B | amide binding |
| GRPR | amide binding |
| GUCY2C | amide binding |
| HADHA | amide binding |
| HCRTR1 | amide binding |
| HCRTR2 | amide binding |
| HFE | amide binding |
| HLA-A | amide binding |
| HLA-B | amide binding |
| HLA-C | amide binding |
| HLA-DPA1 | amide binding |
| HLA-DPB1 | amide binding |
| HLA-DQA1 | amide binding |
| HLA-DQB1 | amide binding |
| HLA-DRA | amide binding |
| HLA-DRB1 | amide binding |
| HLA-DRB3 | amide binding |
| HLA-DRB5 | amide binding |
| HLA-E | amide binding |
| HLA-F | amide binding |
| HLA-G | amide binding |
| HLA-H | amide binding |
| HSP90AB1 | amide binding |
| HSPG2 | amide binding |
| IAPP | amide binding |
| IGF1R | amide binding |
| INHBA | amide binding |
| INSR | amide binding |
| IPO4 | amide binding |
| IPO5 | amide binding |
| ITM2A | amide binding |
| ITM2B | amide binding |
| ITM2C | amide binding |
| IZUMO1R | amide binding |
| KCNIP2 | amide binding |
| KDELR1 | amide binding |
| KDELR2 | amide binding |
| KDELR3 | amide binding |
| KIR3DL1 | amide binding |
| KPNA1 | amide binding |
| KPNA2 | amide binding |
| KPNA3 | amide binding |
| KPNA4 | amide binding |
| KPNA5 | amide binding |
| KPNA6 | amide binding |
| KPNA7 | amide binding |
| KPNB1 | amide binding |
| LANCL1 | amide binding |
| LAPTM4B | amide binding |
| LBP | amide binding |
| LDLR | amide binding |
| LDLRAD3 | amide binding |
| LDLRAP1 | amide binding |
| LEPR | amide binding |
| LILRB1 | amide binding |
| LILRB2 | amide binding |
| LILRB3 | amide binding |
| LRP1 | amide binding |
| LRP8 | amide binding |
| LRPAP1 | amide binding |
| MAG | amide binding |
| MAML1 | amide binding |
| MAP1LC3B | amide binding |
| MAPK8IP2 | amide binding |
| MARCO | amide binding |
| MAS1 | amide binding |
| MC3R | amide binding |
| MC4R | amide binding |
| MCHR1 | amide binding |
| MRGPRX2 | amide binding |
| MSR1 | amide binding |
| NAA80 | amide binding |
| NFATC1 | amide binding |
| NFKBIA | amide binding |
| NGFR | amide binding |
| NKTR | amide binding |
| NLGN1 | amide binding |
| NLRP6 | amide binding |
| NMUR1 | amide binding |
| NMUR2 | amide binding |
| NPAP1 | amide binding |
| NPBWR1 | amide binding |
| NPBWR2 | amide binding |
| NPFFR1 | amide binding |
| NPFFR2 | amide binding |
| NPR2 | amide binding |
| NPR3 | amide binding |
| NPY4R2 | amide binding |
| NQO2 | amide binding |
| NUP153 | amide binding |
| NUP214 | amide binding |
| NUP58 | amide binding |
| NUP98 | amide binding |
| OMP | amide binding |
| OPRD1 | amide binding |
| OPRL1 | amide binding |
| OPRM1 | amide binding |
| PANK1 | amide binding |
| PANK3 | amide binding |
| PC | amide binding |
| PCCA | amide binding |
| PEX19 | amide binding |
| PEX5 | amide binding |
| PEX5L | amide binding |
| PEX7 | amide binding |
| PFDN1 | amide binding |
| PFDN2 | amide binding |
| PFDN4 | amide binding |
| PFDN5 | amide binding |
| PFDN6 | amide binding |
| PGRMC1 | amide binding |
| PHB2 | amide binding |
| PIK3R1 | amide binding |
| PITPNA | amide binding |
| PLA2G4A | amide binding |
| PLEKHA3 | amide binding |
| PLEKHA8 | amide binding |
| PLEKHA8P1 | amide binding |
| PLTP | amide binding |
| PNPLA3 | amide binding |
| POM121 | amide binding |
| POM121B | amide binding |
| POM121C | amide binding |
| POM121L12 | amide binding |
| POM121L2 | amide binding |
| PPARG | amide binding |
| PPIA | amide binding |
| PPIAL4A | amide binding |
| PPIAL4C | amide binding |
| PPIAL4D | amide binding |
| PPIAL4E | amide binding |
| PPIAL4F | amide binding |
| PPIAL4G | amide binding |
| PPIAL4H | amide binding |
| PPIB | amide binding |
| PPIC | amide binding |
| PPID | amide binding |
| PPIE | amide binding |
| PPIF | amide binding |
| PPIG | amide binding |
| PPIH | amide binding |
| PPIL1 | amide binding |
| PPIL2 | amide binding |
| PPIL6 | amide binding |
| PPP3CA | amide binding |
| PPP3R1 | amide binding |
| PPWD1 | amide binding |
| PRLR | amide binding |
| PSAP | amide binding |
| PTGDR2 | amide binding |
| PTGES | amide binding |
| PTH1R | amide binding |
| PTH2R | amide binding |
| RAMP1 | amide binding |
| RAMP2 | amide binding |
| RAMP3 | amide binding |
| RANBP2 | amide binding |
| RANBP6 | amide binding |
| RTN4R | amide binding |
| RXRA | amide binding |
| RYR2 | amide binding |
| SCARB1 | amide binding |
| SCP2 | amide binding |
| SCTR | amide binding |
| SEC61A1 | amide binding |
| SEC61A2 | amide binding |
| SLC40A1 | amide binding |
| SOAT1 | amide binding |
| SOAT2 | amide binding |
| SRD5A1 | amide binding |
| SRD5A2 | amide binding |
| SRP14 | amide binding |
| SRP54 | amide binding |
| SRP68 | amide binding |
| SSTR1 | amide binding |
| SSTR2 | amide binding |
| SSTR3 | amide binding |
| SSTR4 | amide binding |
| SSTR5 | amide binding |
| TAF1 | amide binding |
| TAP1 | amide binding |
| TAP2 | amide binding |
| TIMM22 | amide binding |
| TLR1 | amide binding |
| TLR2 | amide binding |
| TLR4 | amide binding |
| TLR6 | amide binding |
| TM2D1 | amide binding |
| TMEM158 | amide binding |
| TNPO1 | amide binding |
| TNPO2 | amide binding |
| TOMM20 | amide binding |
| TOMM20L | amide binding |
| TOMM40L | amide binding |
| TOMM70 | amide binding |
| TRAV12-1 | amide binding |
| TRAV12-2 | amide binding |
| TRAV12-3 | amide binding |
| TRAV19 | amide binding |
| TRAV23DV6 | amide binding |
| TRAV29DV5 | amide binding |
| TRAV8-4 | amide binding |
| TRBV12-3 | amide binding |
| TRBV28 | amide binding |
| TRBV7-9 | amide binding |
| TREM2 | amide binding |
| TRGV3 | amide binding |
| TRGV9 | amide binding |
| UROS | amide binding |
| VBP1 | amide binding |
| VDAC1 | amide binding |
| VDAC2 | amide binding |
| VIPR1 | amide binding |
| VIPR2 | amide binding |
